# Developing and evaluating a lay health worker delivered implementation intervention to decrease engagement disparities in behavioural parent training: a mixed methods study protocol

**DOI:** 10.1136/bmjopen-2019-028988

**Published:** 2019-07-18

**Authors:** Miya Barnett, Jeanne Miranda, Maryam Kia-Keating, Lisa Saldana, John Landsverk, Anna S Lau

**Affiliations:** 1 Counseling, Clinical, and School Psychology, University of California Santa Barbara, Santa Barbara, California, USA; 2 Psychiatry and Biobehavioral Sciences, University of California Los Angeles, Los Angeles, California, USA; 3 Center for Health Services and Society, University of California Los Angeles, Los Angeles, California, USA; 4 Oregon Social Learning Center, Eugene, Oregon, USA; 5 Psychology, University of California Los Angeles, Los Angeles, California, USA

**Keywords:** lay health workers, implementation strategies, behavioural parent training, mental health disparities

## Abstract

**Introduction:**

Behavioural parent training (BPT) programmes are effective in preventing and treating early-onset conduct problems and child maltreatment. Unfortunately, pervasive mental health service disparities continue to limit access to and engagement in these interventions. Furthermore, challenges with parental engagement can impede the successful implementation of evidence-based practices (EBPs) in community settings that serve low-income, ethnic minority families. Lay health workers (LHWs)—individuals without formal mental health training—represent an important workforce to increase engagement, as they are members of the communities they serve. However, the mobilisation of LHWs has not been well studied as an implementation strategy to extend the reach or effectiveness of EBPs in the USA. LHW-delivered implementation interventions that specifically support the engagement of Latinx parents in evidence-based BPT programmes have the potential to improve clinical and implementation outcomes.

**Methods and analysis:**

A community-partnered approach will use the Quality Implementation Framework (QIF) to tailor and implement an LHW-delivered implementation intervention that aims to promote Latinx parent engagement in BPT programmes. Steps from the QIF will guide study activities to (1) conduct a mixed methods needs assessment to fit the implementation intervention to the local context, (2) adapt LHW-delivered implementation strategies to promote parent access to and engagement in Parent-Child Interaction Therapy and (3) conduct a hybrid effectiveness-implementation pilot trial to examine the feasibility, acceptability and preliminary effectiveness of the LHW implementation intervention at increasing engagement.

**Ethics and dissemination:**

Study procedures have been approved by the Institutional Review Board at the University of California, Santa Barbara. Results will be shared with the community-advisory group, at community-based meetings for other stakeholders involved in the pilot project, and submitted for publication in peer-reviewed journals.

**Strengths and limitations of this study**

- This study seeks to develop and test an implementation intervention to address the impact of underutilisation and poor engagement in behavioural parent training (BPT) programmes, which limit their clinical effectiveness and successful implementation and sustainment.
- This study aims to improve mobilisation of lay health workers, who may be able to offer cultural and linguistic bridges to reach diverse families, as a potential solution to address racial/ethnic disparities in engagement in BPT programmes.
- As a pilot, this study is limited in its sample size to determine the effectiveness of the implementation intervention.
- This study will be limited in its generalisability due to the small sample size, the focus on one BPT programme (Parent-Child Interaction Therapy) and the characteristics of the local context.

## Introduction

Early-onset conduct problems and child maltreatment have been shown to have enormous personal and societal costs, including long-term mental health and substance abuse problems, higher service utilisation and future abuse against women and children.^1–3^ Given that behavioural parent training (BPT) programmes have been shown to be effective at preventing and treating both child maltreatment^4^ and conduct problems^5^ for racially and ethnically diverse families,^6 7^ large systems of care have invested millions of dollars in the implementation of these interventions.^8 9^ Even with major implementation efforts, challenges remain with engagement and retention of families in BPT programmes.^10 11^ A systematic review of engagement in BPT programmes found that at least 25% of families that are appropriate for BPT programmes do not enrol in treatment, and an additional 26% begin, but then drop out of treatment, with higher rates of attrition for low-socioeconomic status families.^12^ In fact, in community implementation of BPT programmes, attrition rates can exceed 65%.^13–15^

The consequences of poor participation in BPT programmes are significant. Families who drop out of treatment are less likely to experience improvements in parenting skills or child disruptive behaviours.^16^ Moreover, failed efforts to recruit and retain parents are costly for providers.^17^ Frequent cancellations and no-shows leads to fewer billable hours for community agencies, which are often under immense financial pressure.^17 18^ Further, inadequate referrals negatively impact the implementation of evidence-based practice (EBP), as therapists may not learn to deliver the practice with fidelity.^8 9 19^ Challenges with engagement may be especially pronounced for racial and ethnic minority families, as mental health service disparities have been well documented.^20^ For example, African–American and Latinx children are almost 50% less likely than non-Latinx, white children to receive treatment for externalising disorders.^21^

In order to meet the public health potential of BPT programmes and address service disparities, implementation interventions are needed to support parental engagement for ethnic minority parents. Implementation interventions, which are usually complex and multilevel, include strategies to enhance the adoption and ongoing implementation of clinical interventions at the organisation, provider and consumer levels.^22^ Multiple implementation strategies have been identified that focus on increasing consumer engagement with EBPs, including (1) increasing demand for EBPs, (2) intervening with consumers to enhance uptake and adherence and (3) preparing consumers to be active participants in treatment.^23^ These implementation strategies are consistent with evidence-based approaches to improve engagement in children’s mental healthcare, which include assessment of barriers, accessibility promotion, psychoeducation about services and appointment reminders.^24 25^

### Addressing mental health service disparities

Lay health workers (LHWs) may be especially well positioned to deliver consumer-facing implementation strategies focused on addressing service disparities for underserved, low-resource communities.^26^ LHWs, which include a range of terms, including *promotores*, family peer advocates and wellness navigators, are individuals without formal mental health training, who have roles intended to increase their community’s access to and benefit from services.^27 28^ LHWs have the potential to address both demand and supply drivers of disparities in EBP delivery.^26^ Demand for EBPs is impacted by an individual’s mental health literacy, stigma towards mental illness and help seeking, perceptions of treatment providers and culturally based beliefs and preferences.^29 30^ Systemic barriers to care may exacerbate disparities in accessing care. For example, undocumented immigrants are especially unlikely to seek mental health services due to fear of being reported to authorities.^31^ Since LHWs come from similar cultural and personal backgrounds as the individuals they serve, they may be especially adept at helping patients overcome distrust of health systems.^32^

Regarding supply, the number of professional mental health providers who can deliver linguistically and culturally competent EBPs is inadequate.^33^ The majority of mental health research with LHWs has been conducted in low-income and middle-income countries, with emerging evidence that LHWs can improve mental health outcomes when they are tasked with delivering EBPs.^28 34 35^ Although LHWs have successfully delivered BPT programmes as prevention interventions in high-income countries, using a task-shifting model,^36–38^ licensure and certification requirements frequently restrict EBP delivery to professionals in mental health settings.^26^ Therefore, LHWs in the USA may need to have complementary and distinct roles within the provision of EBPs.^11 26^ For example, if LHWs delivered auxiliary engagement services (eg, outreach and case management), it could reduce the burden on bilingual and bicultural mental health professionals and allow them to focus on activities that require advanced training and licensure, such as providing EBPs for more clients.^19 39^

One example of an LHW-delivered engagement programme is the Parent Empowerment Programme (PEP), which trains family peer advocates to work with parents to address their children’s mental health needs and overcome barriers to care.^40 41^ The majority of research on PEP has focused on evaluating the training of family peer advocates, as opposed to investigating the impact of the model on clinical outcomes for families, service utilisation or engagement in EBPs.^40–43^ One randomised control trial evaluated the impact of PEP for black and Latinx parents of children with autism. Parents who received PEP had significantly lower stress than parents who received treatment as usual. However, there were no group differences for service utilisation. The researchers advocated that future research on programmes such as PEP should include non-English-speaking families, who may have higher levels of need, and use qualitative research to better understand the strengths and areas of improvement for the model.^44^ The proposed study follows these recommendations through the development and evaluation of LHWs Enhancing Engagement for Parents (LEEP), an implementation intervention to improve engagement for low-income, Latinx parents into one BPT programme, Parent-Child Interaction Therapy (PCIT).^45^ LEEP seeks to follow recommendations by Chacko and colleagues^12^ based on their systematic review of parental engagement in BPT programmes by ‘preparing parents for BPT, addressing practical barriers to engagement, assisting in aligning parent’s involvement with their own goals for treatment’ (p. 211) in order to impact initial and ongoing engagement in PCIT.

### Parent-child interaction therapy

PCIT has unique benefits and challenges related to engaging parents in treatment. The treatment model uses in vivo feedback to overcome challenges that are inherent to teaching methods used in other BPT programmes (eg, didactics, discussion) as it necessitates active participation and assesses learning in real time. PCIT requires that parents demonstrate a high level of proficiency with the targeted parenting skills before they advance from the first phase of treatment, which focuses on enhancing the parent-child relationship, to the second phase of treatment, which teaches effective and developmentally appropriate limit setting and discipline approaches, and then until they graduate from treatment.^45^ Mastery-based criteria guarantee that all parents can successfully use the skills; however, parents often drop out before they learn the full range of parenting skills needed to decrease disruptive behaviours.^13 16^ Furthermore, some research suggests that low-income, ethnic minority parents require more practice and time in treatment to reach this level of skill proficiency.^46–48^ Extended treatment length can lead to long waitlists and fewer families seen in PCIT.^49^ Further, problems with attendance, retention and prolonged skill acquisition have downstream effects on PCIT provider implementation outcomes. Clinicians can take up to 3 years to meet PCIT certification requirements (ie, achieving fidelity and graduating two cases).^50^ Thus, low parental engagement results in provider attrition from training, which in turn compromises the sustainability of the intervention and limits the return on costly investments made to implement PCIT in public service systems.^8 9^

**Figure 1:**
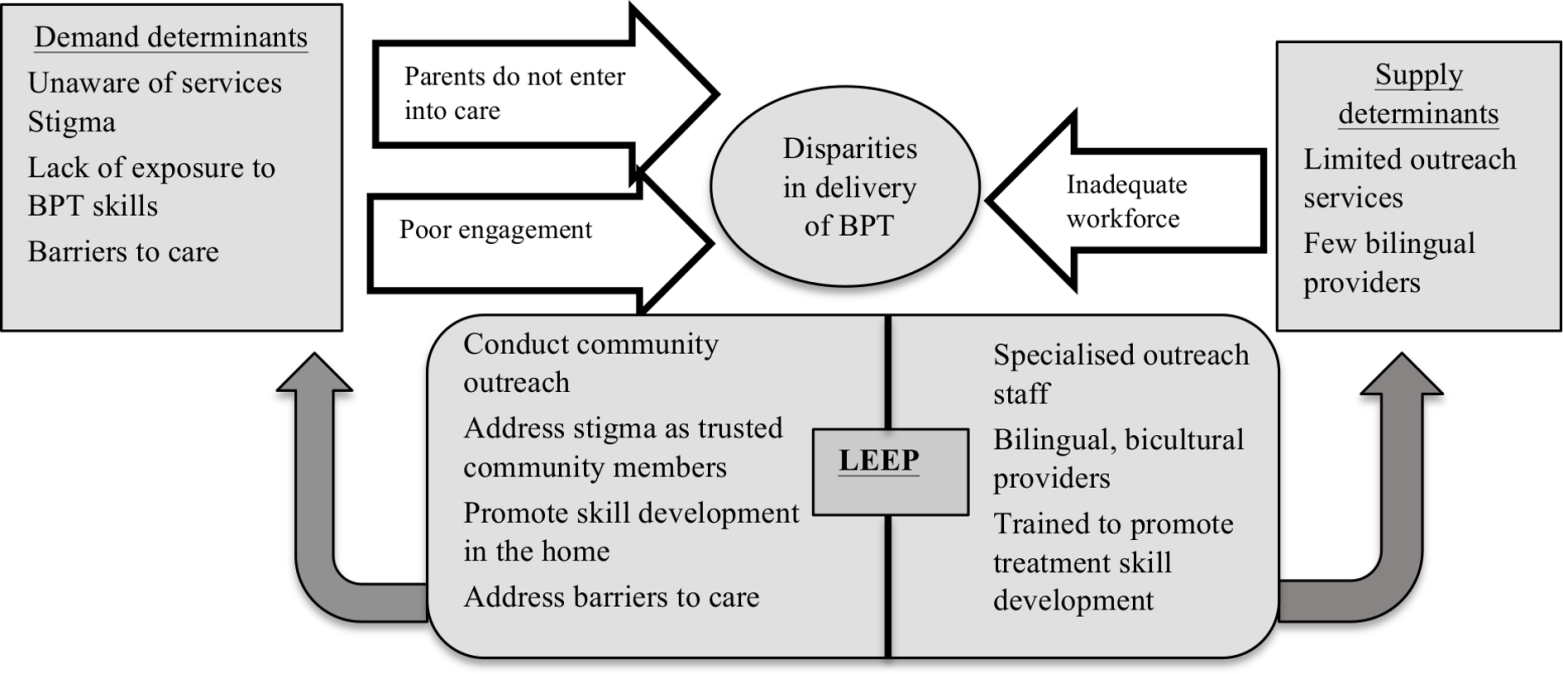
LEEP’s approach to address supply and demand determinants of disparities. Adapted from Barnett *et al*.^26^ BPT, behavioural parent training programme; LEEP, LHW Enhancing Engagement for Parents; LHW, lay health workers.

LEEP seeks to improve the supply of and demand for PCIT in agencies that predominately serve low-income, Latinx immigrant families, and address engagement challenges that impact clinical and implementation outcomes (figure 1). PCIT is widely implemented in community settings, including in the county where the current study is being conducted. LEEP includes LHW-delivered implementation strategies to increase caregiver engagement as an extension of PCIT services, which will be provided by the licensed mental health professionals. LEEP will be compared with PCIT implementation-as-usual to see if parental engagement and implementation challenges are ameliorated. A community-partnered approach will focus on making LEEP a feasible and acceptable implementation intervention, with the following aims.

#### Aim 1

Assess the current context of LHW mobilisation in children’s mental health services, to inform the development of LEEP.

#### Aim 2

Through community partnership, develop a structure for the implementation of LEEP in publicly funded, children’s mental health settings.

#### Aim 3

Evaluate the feasibility of implementing LEEP in community mental health agencies through a pilot effectiveness-implementation trial.

## Methods and analysis

### Conceptual framework and approach

**Figure 2:**
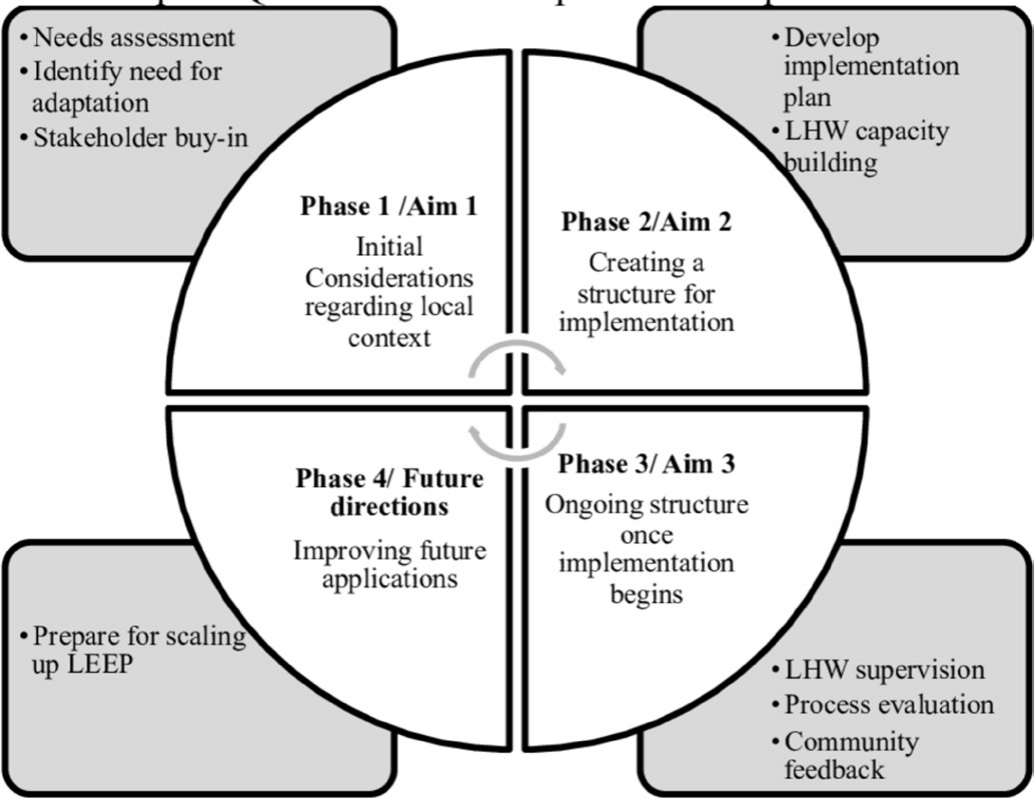
Critical steps of QIF in LEEP development and implementation. Adapted from Meyers *et al*.^51^ LEEP, LHWs Enhancing Engagement for Parents; LHW, lay health worker.

The Quality Implementation Framework (QIF), which includes four phases to support high quality implementation, informs the study aims and plans for scaling-up LEEP (figure 2).^51^ The first phase of the QIF focuses on assessing organisational needs, readiness and innovation-organisational fit, which will be conducted in aim 1 through survey data and stakeholder interviews. Phase 2 in the QIF focuses on the development of implementation structures, which will occur during the second aim with the input of a community-advisory group. The community-advisory group will collaboratively help to develop an implementation plan delineating tasks and timelines to establish infrastructure for LHW capacity building, including job descriptions and training plans. Phase 3 of the QIF includes three main activities that will take place during a hybrid type 2 effectiveness-implementation pilot stepped-wedge trial of LEEP. These activities include (1) providing implementation support strategies (eg, supervision and consultation) to LHWs, (2) conducting a process evaluation to identify successes and barriers to implementation and (3) providing ongoing feedback to organisations about the impact LEEP is having on service outcomes. Finally, aim 3 activities will inform phase 4 of the QIF, which focuses on learning from initial implementation experiences to inform future efforts to scale-up and sustain LEEP.

### Patient and public involvement

The research questions, study design and outcomes measures were informed by public stakeholders, including agency leaders, PCIT therapists and LHWs. No patients were involved in this process, although LHWs often have shared characteristics and life experiences given their social proximity to the individuals in the communities they serve.^52^ The burden of the randomised control trial will be evaluated through the collection of feasibility and acceptability data in qualitative interviews with participants. Results will be shared with participants in community-based events.

#### Aim 1

Assess the current context of LHW mobilisation in children’s mental health services, to inform the development of LEEP.

##### Participants

Surveys will be administered to LHWs employed or contracted by children’s mental health agencies in two counties in California. Approximately 70 LHWs will be recruited to complete the quantitative survey. Based on a national survey of LHW,^53^ LHWs are expected to be Latinx, female and have below a college level of education. Ten agency community mental health agency leaders and 25–30 LHWs will be invited to participate in interviews to expand on the findings from the survey. Survey and interviews will be offered in Spanish or English.

##### Procedure

A mixed method needs assessment will be conducted to understand how LHWs are currently mobilised in children’s community mental health settings, with the purpose of adapting LEEP to fit within the local context. Surveys will provide a breadth of information and qualitative interviews will provide depth of information, to understand perceived barriers to parental engagement in children’s mental health services, LHW roles and integration into services, and LHW knowledge about and attitudes towards BPT programmes and evidence-based engagement strategies. Data collection started in January 2017 and was completed in December 2018.

##### Survey measures

Surveys will be collected via electronic or paper-and-pencil survey based on LHW preferences.

**LHW characteristics** 39 A demographic questionnaire will provide information about the LHWs’ characteristics, including gender, race/ethnicity, country of origin, educational level and years of experience.

**Cultural Background Questionnaire** 54 The Cultural Background Questionnaire is a 19-item self-report measure used to assess therapist generational status and acculturation, including cultural identity (ie, US identity and Heritage Cultural Identity) and language use.

**Parental Engagement** 55 56 A questionnaire that was developed to measure provider’s perceptions of and strategies for engaging fathers has been adapted to measure LHW’s perceptions of barriers to engagement, strategies for engagement and confidence in engagement for parents. The adapted questionnaire includes perceived barriers in engagement for parents in general, and the LHW’s use of and confidence with engagement strategies for both mothers and fathers.

**Attitudes towards BPT strategies.** A four-item questionnaire was developed for this study to measure LHW’s attitudes towards teaching parents common strategies targeted in BPT programmes including play to improve the parent-child relationship, praise of positive behaviours, ignoring minor misbehaviours and time-out as a form of discipline.

**EBP Questionnaire** 57 A questionnaire developed to measure service broker’s knowledge of and referrals to EBPs has been adapted to identify if LHWs are aware of, making referrals to and supporting families involved specifically in PCIT(eg, *‘Have you referred parents to Parent-Child Interaction Therapy (PCIT)’)*.

##### Semistructured Interviews

Interview guides will include topics, questions and probes related to LHW roles, training needs and experiences and attitudes related to BPT programmes. Questions will investigate how LHWs view their roles in agencies and their communities, their perceptions of BPT programmes, their preparation for their position and their training needs. Interviews with agency leaders will focus on how LHWs are integrated into services, LHW training, and outreach and engagement strategies with Latinx families. A ‘funnel-approach’ will be used with broad open-ended questions related to roles, trainings and attitudes asked first, followed with specific probes to elicit details.^58^

##### Analysis

A QUAN+QUAL mixed methods design will be used, with quantitative and qualitative data collected simultaneously and given equal weight in analyses, for the purposes of gaining breadth and depth of understanding (ie, complimentarity), identifying if the qualitative and quantitative data provide the same answer to the same question (ie, convergence), and using qualitative data expand on unexpected quantitative findings.^59^ Interviews will be transcribed and entered into NVivo, a software that aids the coding, organisation and retrieval of codes. An iterative process will be used where the coding team first develops a preliminary coding scheme and applies it to a sample text to ensure all relevant themes are captured. Once a final coding scheme is decided on, coders will apply the final code list to all transcripts. Regular meetings with the coding team will be conducted to examine coding across analysts, resolve differences in coding, conduct iterative refinement of code definitions and the logic of the coding tree, and collaborate on the development of themes. Qualitative themes will be identified through analysis of co-occurring codes and text analysis.^60 61^

#### Aim 2

Through community partnership, develop a structure for the implementation of LEEP in children’s mental health settings.

##### Participants

A community-advisory group with six to nine stakeholders will be formed to make sure that implementation supports match the local context. Agency leaders, PCIT therapists and LHWs will be represented in the advisory group. Given the wide diversity of viewpoints, education levels and ethnicities, efforts will be made to provide each participant with equal representation, opportunities for contribution and honorariums.

##### Procedure

In line with the Model of Research-Community Partnership, which was specifically developed for research in children’s mental health services, the formation of the partnership will focus on building relationships, trust, establishing a joint mission and identifying roles and responsibilities of different partner members. This will provide the foundation to build a synergistic, collaborative relationship focused on developing and delivering LEEP, which in turn could improve the successful and sustained implementation of PCIT.^62^ Using data from aim 1 and in collaboration with the community-advisory group, the LEEP implementation intervention will be adapted from an existing protocol focused on LHW-delivered parent outreach and engagement strategies. This protocol was developed to increase access to PCIT in a low-income, Latinx community in the Southeastern USA, but has not been disseminated to other communities.^39^ The implementation supports needed for LHWs to deliver LEEP also will be identified and put into place. Steps from the QIF will be used to guide the activities of the community-advisory group in adapting these materials and developing LEEP’s implementation structure.^51^ Advisory group meetings will include (1) activities to build trust and develop a shared mission statement, (2) feedback on adapting LEEP materials, (3) advisory group input on survey and interview results from aim 1 and (4) development of a plan with specific tasks, roles, tracking for LEEP implementation. Phase 2 activities involving the community-advisory group began in December 2018 and will continue through March 2021.

#### Aim 3

Evaluate the feasibility of implementing LEEP through a pilot effectiveness-implementation trial.

In aim 3, an effectiveness/implementation hybrid design (type 2) pilot study will integrate qualitative and quantitative data to examine the feasibility of delivering and scaling-up LEEP. Type 2 hybrid trials simultaneously measure the clinical effectiveness of an intervention, in this case PCIT, and the feasibility and utility of an implementation intervention (ie, LEEP).^63^ Pilot studies are limited in their ability to test effectiveness given small sample sizes, but they provide a critical phase of research design that can examine the feasibility of the approach to be used in a large-scale study.^64^ A focus will be placed on measuring engagement outcomes at a client and agency levels to evaluate if LEEP is increasing the reach of PCIT services, service entry and treatment engagement.

##### Procedure

**Figure 3:**
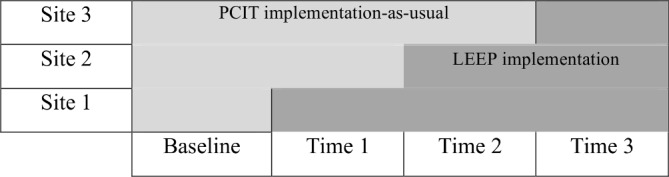
Stepped-wedge trial of LEEP implementation. A stepped-wedge design will be used with the three sites implementing LEEP at separate time points. LEEP, LHWs Enhancing Engagement for Parents; LHW, lay health worker; PCIT, Parent-Child Interaction Therapy.

Three agency sites that provide PCIT will be involved in this pilot study, which will use a stepped-wedge design. In a stepped-wedge design, a period of baseline measurement will occur for all sites, in which PCIT will be implemented-as-usual. Then at subsequent time points, each site will be randomised to LEEP and response to the intervention will be measured for client and implementation outcomes (figure 3). At each agency, one to two LHWs (four to six in total) will be trained to deliver LEEP. Client and implementation outcomes will be collected during PCIT implementation-as-usual and LEEP implementation. The baseline measurement period began in January 2019. LHWs will be trained to deliver LEEP at the first site starting in July 2019 and will continue through March 2021.

##### Participants

Four to six LHWs will be trained to provide LEEP. These LHWs will provide LEEP care extension services to approximately four PCIT clients each (16–24 families). LHWs will be trained to provide informed consent for parents. Families that participate in LEEP or PCIT implementation-as-usual will meet criteria for receiving this BPT programme. This includes having a child between the ages of 2 and 7 and presenting problems consistent with disruptive behaviours, or risk for child maltreatment.

##### LEEP intervention

Based on community-advisory group feedback on the needs of the Latinx immigrant community and the agencies implementing PCIT, along with research on parental engagement,^11^ LEEP includes components for LHWs to (1) increase awareness of PCIT for Latinx immigrant families, (2) promote engagement once parents seek PCIT services and (3) support parents’ use of skills taught in PCIT throughout treatment. To increase knowledge of PCIT, LHWs will conduct community presentations in locations with parents of young children (eg, Head Start Centres, churches). Parents will be referred to LEEP when they first seek services to promote engagement. LHWs will meet with parents in their home to discuss identify how PCIT aligns with their goals for treatment, address practical barriers to engagement (eg, transportation) and introduce the relationship-enhancing parenting skills taught in PCIT. Once parents start PCIT, LHWs will provide home visits to promote skill practice and treatment adherence at the beginning of each treatment phase. Additional booster sessions will be provided based on the parent’s progress in treatment. If parents have not reached mastery criteria after five sessions, LHWs will conduct weekly home visits to reinforce skill use and address barriers to engagement. LHWs will be provided with electronic tablets with e-books that included scripts and videos to use in one-on-one meetings with parents before they enter into care and while they are receiving PCIT services. The e-books will have materials to help LHWs promote motivation (eg, parent testimonials), homework adherence and skill practice (eg, video demonstrations of the targeted parenting skills).

##### Effectiveness outcome measures

Given that PCIT is an assessment-driven BPT programme, clinical outcomes will be assessed using standard measures that are collected as part of the routine PCIT protocol. Parents will not complete any additional measures for this study.

*Engagement*. To assess if LEEP impacts engagement at the family level, session attendance, graduation from PCIT and the number of sessions needed to graduate will be assessed. Further, daily skill practice will be measured using the record sheets that parents complete over the week, which has been used in the past studies on homework adherence.^65^

*The Dyadic Parent-Child Interaction Coding System (DPICS)*.^66^ The DPICS is a behavioural observation coding system that was designed to measure the quality of interaction in parent-child dyads, which has good inter-rater reliability. This study will use the DPICS categories, *Behaviour Description, Labelled Praise, Unlabelled Praise, Reflection, Question, Negative Talk, and Indirect and Direct Commands*, to measure the parent’s skill acquisition.

*Eyberg Child Behaviour Inventory (ECBI)*.^67^ The ECBI is a 36-item parent-rating scale of disruptive behaviour problems for children between the ages of 2 and 16. Parents rate the frequency of each disruptive behaviour on a seven-point Likert scale ranging from *never* (1) to *always* (7), which are summed to yield the Intensity Scale and whether this behaviour is a problem for them, with the total number of yes responses yielding the Problem Scale.

##### Implementation outcomes

Using the implementation outcomes outlined by Proctor and colleagues,^68^ this study uses mixed methods to understand the acceptability, appropriateness, feasibility, reach and costs of delivering LEEP.

##### LHW level outcomes

To measure changes in LHW knowledge, perceptions of acceptability and feasibility of PCIT, and competence, LHW will complete pre-self-report and post-self-report and behavioural measures (table 1). Ongoing fidelity monitoring will be conducted throughout LHW’s delivery of LEEP. Fidelity monitoring will include reviewing data capture of the ebook created for LEEP, which will include videos about PCIT and scripts for the LHWs to use with the families they serve. LHWs will also audio record their sessions to monitor fidelity to the LEEP model.

**Table 1:** Measures of LHW training and outcomes

Measure	Description	Administration
*Demographic and Background Form* 39 54	Characterises personal and professional backgrounds of LHW	Aim 1: survey Aim 3: training
*EBP Questionnaire* 57	Measures service brokers’ awareness of and referrals to EBPs.	Aim 1: survey
*Parent Engagement Strategy Use and Confidence* 55 56	Assess use and confidence of engagement strategies with mothers and fathers.	Aim 1: survey Aim 3: pretraining, post-training
*Acceptability and Feasibility of PCIT* 69	Assess LHW perceptions of the acceptability and feasibility of PCIT	Aim 1: survey Aim 3: pretraining, post-training
*PCIT Knowledge Quiz* 39	A quiz that measures the knowledge of PCIT principles and practices	Aim 3: pretest, post-training
*Dyadic Parent-Child Interaction Coding System* 66	A behavioural observation coding system that assesses parent-child interactions. It will be used to measure LHW’s ability to model parenting behaviours.	Aim 3: pretraining, post-training
*LEEP Fidelity Monitoring*	Review of ebook data capture to see the LEEP resources being used and audio recordings of home sessions.	Aim 3: during consultation

EBP, evidence-based practice; LEEP, LHWs Enhancing Engagement for Parents; LHW, lay health worker; PCIT, Parent-Child Interaction Therapy.

##### Implementation costs

Costs associated with delivering LEEP will be measured by calculating time estimates associated with all aspects of implementation.

##### Agency efficiencies

To identify if LEEP impacts agency efficiencies, with therapists increasing their billable hours, administrative claims will be calculated for PCIT therapists to measure the time spent in direct services.

##### Reach and penetration

At the agency level, reach of PCIT will be assessed by the number of clients that enrol in and graduate from PCIT. Using administrative claims data, penetration at the agency level will be calculated as the percentage of children receiving PCIT in out of the number of children who are eligible for this EBP. Furthermore, the percentage of families that successfully complete PCIT out of the families enrolled will be calculated.

##### Acceptability and feasibility

Qualitative interviews will be conducted with the LHWs, agency leaders and 10 parents to assess their perceptions of LEEP including perceived acceptability, appropriateness and feasibility, which are important early implementation outcomes.^68^

##### Analysis

This pilot trial is designed to evaluate the feasibility of implementing LEEP and develop tools to measure its clinical and implementation targets and outcomes. The trial is not powered to assess intervention effects. Analyses will focus on establishing the reliability and validity of measures of clinical engagement and implementation outcomes. Qualitative data will be analysed using the methodology described in aim 1. Qualitative and quantitative data will be given equal weight in analyses with a focus on *convergence*, *expansion* and *complimentarity*, with quantitative data used to measure outcomes and qualitative data to understand process.^59^

## Discussion

LEEP has the potential for a significant public health impact, by developing an implementation intervention to increase entry and engagement of Latinx parents into BPT programmes to improve clinical and implementation outcomes. Although LHWs have been identified as an important workforce to address mental health disparities, limited research has evaluated the best strategies to mobilise them to support EBP implementation in the USA.^28^ As a pilot study, findings will be limited in power and generalisability. However, the exploratory and development work in this study will provide data on the feasibility and acceptability of LEEP and its preliminary impact on client recruitment, adherence and retention in PCIT, which will inform future scaling-up of the model.

### Ethics and dissemination

Study procedures have been approved by the Institutional Review Board at the University of California, Santa Barbara. Results will be submitted for publication in peer-reviewed journals. Furthermore, results will be shared with the community-advisory board and other stakeholders involved in the pilot of LEEP.

## Supplementary Material

Reviewer comments

Author's manuscript

## References

[1] Fergusson DM, Horwood LJ, Ridder EM Show me the child at seven: the consequences of conduct problems in childhood for psychosocial functioning in adulthood. J Child Psychol Psychiatry 2005;46:837–49. 10.1111/j.1469-7610.2004.00387.x 16033632

[2] Rivenbark JG, Odgers CL, Caspi A, et al The high societal costs of childhood conduct problems: evidence from administrative records up to age 38 in a longitudinal birth cohort. J Child Psychol Psychiatry 2018;59:703–10. 10.1111/jcpp.12850 29197100 PMC5975095

[3] Prinz RJ, Sanders MR, Shapiro CJ, et al Addendum to "population-based prevention of child maltreatment: the U.S. Triple P System Population Trial". Prev Sci 2016;17:410–6. 10.1007/s11121-016-0631-x 26780665

[4] Chen M, Chan KL Effects of parenting programs on child maltreatment prevention: a meta-analysis. Trauma Violence Abuse 2016;17:88–104. 10.1177/1524838014566718 25573846

[5] Kaminski JW, Claussen AH Evidence base update for psychosocial treatments for disruptive behaviors in children. J Clin Child Adolesc Psychol 2017;46:477–99. 10.1080/15374416.2017.1310044 28459280 PMC5600477

[6] Miranda J, Bernal G, Lau A, et al State of the science on psychosocial interventions for ethnic minorities. Annu Rev Clin Psychol 2005;1:113–42. 10.1146/annurev.clinpsy.1.102803.143822 17716084 PMC4470614

[7] Ortiz C, Del Vecchio T Cultural diversity: do we need a new wake-up call for parent training? Behav Ther 2013;44:443–58. 10.1016/j.beth.2013.03.009 23768671

[8] Beveridge RM, Fowles TR, Masse JJ, et al State-wide dissemination and implementation of parent–child interaction therapy (PCIT): Application of theory. Child Youth Serv Rev 2015;48:38–48. 10.1016/j.childyouth.2014.11.013

[9] Timmer SG, Urquiza AJ, Boys DK, et al Filling potholes on the implementation highway: Evaluating the implementation of Parent-Child Interaction Therapy in Los Angeles County. Child Abuse Negl 2016;53:40–50. 10.1016/j.chiabu.2015.11.011 26704299

[10] Baker CN, Arnold DH, Meagher S Enrollment and attendance in a parent training prevention program for conduct problems. Prev Sci 2011;12:126–38. 10.1007/s11121-010-0187-0 21052834

[11] Sanders MR Triple P-Positive Parenting Program as a public health approach to strengthening parenting. J Fam Psychol 2008;22:506–17. 10.1037/0893-3200.22.3.506 18729665

[12] Chacko A, Jensen SA, Lowry LS, et al Engagement in behavioral parent training: review of the literature and implications for practice. Clin Child Fam Psychol Rev 2016;19:204–15. 10.1007/s10567-016-0205-2 27311693

[13] Lanier P, Kohl PL, Benz J, et al Parent–child interaction therapy in a community setting: examining outcomes, attrition, and treatment setting. Res Soc Work Pract 2011;21:689–98. 10.1177/1049731511406551 PMC4021486 24839378

[14] Lyon AR, Budd KS A community mental health implementation of parent-child interaction therapy (PCIT). J Child Fam Stud 2010;19:654–68. 10.1007/s10826-010-9353-z 20877583 PMC2945385

[15] Pearl E, Thieken L, Olafson E, et al Effectiveness of community dissemination of parent–child interaction therapy. Res Pract Policy 2012;413:204–13. 10.1037/a0022948

[16] Boggs SR, Eyberg SM, Edwards DL, et al Outcomes of parent-child interaction therapy: a comparison of treatment completers and study dropouts one to three years later. Child Fam Behav Ther 2005;26:1–22. 10.1300/J019v26n04_01

[17] Hoagwood KE, Olin SS, Horwitz S, et al Scaling up evidence-based practices for children and families in New York State: toward evidence-based policies on implementation for state mental health systems. J Clin Child Adolesc Psychol 2014;43:145–57. 10.1080/15374416.2013.869749 24460518 PMC3954943

[18] Stewart RE, Adams DR, Mandell DS, et al The perfect storm: collision of the business of mental health and the implementation of evidence-based practices. Psychiatr Serv 2016;67:159–61. 10.1176/appi.ps.201500392 26522680 PMC4790728

[19] Regan J, Lau AS, Barnett M, et al Agency responses to a system-driven implementation of multiple evidence-based practices in children’s mental health services. BMC Health Serv Res 2017;17:671 10.1186/s12913-017-2613-5 28927407 PMC5606027

[20] Alegria M, Vallas M, Pumariega AJ Racial and ethnic disparities in pediatric mental health. Child Adolesc Psychiatr Clin N Am 2010;19:759–74. 10.1016/j.chc.2010.07.001 21056345 PMC3011932

[21] Coker TR, Elliott MN, Kataoka S, et al Racial/Ethnic disparities in the mental health care utilization of fifth grade children. Acad Pediatr 2009;9:89–96. 10.1016/j.acap.2008.11.007 19329099 PMC4586149

[22] Eldh AC, Almost J, DeCorby-Watson K, et al Clinical interventions, implementation interventions, and the potential greyness in between -a discussion paper. BMC Health Serv Res 2017;17:16 10.1186/s12913-016-1958-5 28061856 PMC5219812

[23] Powell BJ, Waltz TJ, Chinman MJ, et al A refined compilation of implementation strategies: results from the Expert Recommendations for Implementing Change (ERIC) project. Implement Sci 2015;10:21 10.1186/s13012-015-0209-1 25889199 PMC4328074

[24] Becker KD, Lee BR, Daleiden EL, et al The common elements of engagement in children’s mental health services: which elements for which outcomes? J Clin Child Adolesc Psychol 2015;44:30–43. 10.1080/15374416.2013.814543 23879436

[25] Becker KD, Boustani M, Gellatly R, et al Forty years of engagement research in children’s mental health services: multidimensional measurement and practice elements. J Clin Child Adolesc Psychol 2018;47:1–23. 10.1080/15374416.2017.1326121 28574780

[26] Barnett ML, Lau AS, Miranda J Lay health worker involvement in evidence-based treatment delivery: a conceptual model to address disparities in care. Annu Rev Clin Psychol 2018;14:185–208. 10.1146/annurev-clinpsy-050817-084825 29401043 PMC5940491

[27] Ayala GX, Vaz L, Earp JA, et al Outcome effectiveness of the lay health advisor model among Latinos in the United States: an examination by role. Health Educ Res 2010;25:815–40. 10.1093/her/cyq035 20603384 PMC2948840

[28] Barnett ML, Gonzalez A, Miranda J, et al Mobilizing community health workers to address mental health disparities for underserved populations: a systematic review. Adm Policy Ment Health 2018;45:195–211. 10.1007/s10488-017-0815-0 28730278 PMC5803443

[29] Alegria M, Vallas M, Pumariega A, et al NIH Public Access. Child Youth Serv Rev 2015;44:181–217.

[30] Kilbourne AM, Switzer G, Hyman K, et al Advancing health disparities research within the health care system: a conceptual framework. Am J Public Health 2006;96:2113–21. 10.2105/AJPH.2005.077628 17077411 PMC1698151

[31] Philbin MM, Flake M, Hatzenbuehler ML, et al State-level immigration and immigrant-focused policies as drivers of Latino health disparities in the United States. Soc Sci Med 2018;199:29–38. 10.1016/j.socscimed.2017.04.007 28410759 PMC5632125

[32] Katigbak C, Van Devanter N, Islam NT-S Partners in health: a conceptual framework for the role of community health workers in facilitating patients’ adoption of healthy behaviors. J Public 2015. 10.2105/AJPH.2014.302411 PMC4386525 25790405

[33] Huang L, MacBeth G, Dodge J, et al Transforming the workforce in children’s mental health [Internet]. Vol. 32. Administration and Policy in Mental Health 2004;87:167. 10.1023/b:apih.0000042745.64582.72 15586849

[34] Singla DR, Kohrt BA, Murray LK, et al Psychological Treatments for the World: lessons from low- and middle-income countries. Annu Rev Clin Psychol 2017;13:149–81. 10.1146/annurev-clinpsy-032816-045217 28482687 PMC5506549

[35] Non-specialist health worker interventions for the care of mental, neurological and substance-abuse disorders in low- and middle-income countries SO. Cochrane Database of Systematic Reviews 2013;11. 10.1002/14651858.CD009149.pub2 24249541

[36] Chacko A, Scavenius C Bending the curve: a community-based behavioral parent training model to address adhd-related concerns in the voluntary sector in Denmark. J Abnorm Child Psychol 2018;46:505–17. 10.1007/s10802-017-0310-9 28536873

[37] Chacko A, Fabiano GA, Doctoroff GL, et al Engaging fathers in effective parenting for preschool children using shared book reading: a randomized controlled trial. J Clin Child Adolesc Psychol 2018;47:79–93. 10.1080/15374416.2016.1266648 28103110 PMC5788184

[38] Williamson AA, Knox L, Guerra NG, et al A pilot randomized trial of community-based parent training for immigrant Latina mothers. Am J Community Psychol 2014;53(1-2):47–59. 10.1007/s10464-013-9612-4 24276907

[39] Barnett ML, Davis EM, Callejas LM, et al The development and evaluation of a natural helpers’ training program to increase the engagement of urban, Latina/o families in parent-child interaction therapy. Child Youth Serv Rev 2016:65.28496286

[40] Rodriguez J, Olin SS, Hoagwood KE, et al The development and evaluation of a parent empowerment program for family peer advocates. J Child Fam Stud 2011;20:397–405. 10.1007/s10826-010-9405-4 25382959 PMC4223802

[41] Olin SS, Hoagwood KE, Rodriguez J, et al Impact of empowerment training on the professional work of family peer advocates. Child Youth Serv Rev 2010;32:1426–9. 10.1016/j.childyouth.2010.06.012 21076659 PMC2976547

[42] Olin SS, Williams N, Pollock M, et al Quality indicators for family support services and their relationship to organizational social context. Adm Policy Ment Health 2014;41:43–54. 10.1007/s10488-013-0499-z 23709286 PMC3858410

[43] Hoagwood KE, Olin SS, Storfer-Isser A, et al Evaluation of a train-the-trainers model for family peer advocates in children’s mental health. J Child Fam Stud 2018;27:1130–6. 10.1007/s10826-017-0961-8 29576726 PMC5854741

[44] Jamison JM, Fourie E, Siper PM, et al Examining the efficacy of a family peer advocate model for black and hispanic caregivers of children with autism spectrum disorder. J Autism Dev Disord 2017;47:1314–22. 10.1007/s10803-017-3045-0 28168677

[45] Funderburk BW, Eyberg S Parent–child interaction therapy History of psychotherapy: continuity and change (2nd. Washington: American Psychological Association:415–20.

[46] McCabe K, Yeh M Parent-child interaction therapy for Mexican Americans: a randomized clinical trial. J Clin Child Adolesc Psychol 2009;38:753–9 http://www.ncbi.nlm.nih.gov/pubmed/20183659 10.1080/15374410903103544 20183659

[47] Matos M, Bauermeister JJ, Bernal G Parent-child interaction therapy for Puerto Rican preschool children with ADHD and behavior problems: a pilot efficacy study. Fam Process 2009;48:232–52. 10.1111/j.1545-5300.2009.01279.x 19579907

[48] Ramos G, Blizzard AM, Barroso NE, et al Parent training and skill acquisition and utilization among spanish- and english-speaking latino families. J Child Fam Stud 2018;27:268–79. 10.1007/s10826-017-0881-7 29456439 PMC5813840

[49] Gross D, Belcher HME, Budhathoki C, et al Does parent training format affect treatment engagement? A randomized study of families at social risk? J Child Fam Stud 2018;27:1579–93. 10.1007/s10826-017-0984-1 29713137 PMC5918300

[50] Scudder A, Herschell AD Building an evidence-base for the training of evidence-based treatments in community settings: Use of an expert-informed approach. Child Youth Serv Rev 2015;55:84–92. 10.1016/j.childyouth.2015.05.003 26504259 PMC4617599

[51] Meyers DC, Durlak JA, Wandersman A The quality implementation framework: a synthesis of critical steps in the implementation process. Am J Community Psychol 2012;50(3-4):462–80. 10.1007/s10464-012-9522-x 22644083

[52] Gustafson EL, Atkins M, Rusch D Community health workers and social proximity: implementation of a parenting program in urban poverty. Am J Community Psychol 2018;62(3-4):449–63. 10.1002/ajcp.12274 30222866

[53] Ingram M, Reinschmidt KM, Schachter KA, et al Establishing a professional profile of community health workers: results from a national study of roles, activities and training. J Community Health 2012;37:529–37. 10.1007/s10900-011-9475-2 21964912 PMC6684283

[54] Saifan D, Brookman-Frazee L, Barnett M, et al Ethnic minority community therapists’ acculturation and reported adaptations to children’s evidence-based practices. Cultur Divers Ethnic Minor Psychol 2018;24:530–40. 10.1037/cdp0000203 29963881 PMC6188807

[55] Tully LA, Collins DAJ, Piotrowska PJ, et al Examining practitioner competencies, organizational support and barriers to engaging fathers in parenting interventions. Child Psychiatry Hum Dev 2018;49:109–22. 10.1007/s10578-017-0733-0 28523378 PMC5813069

[56] Jiang Y, Tully LA, Burn MT, et al Development and psychometric evaluation of the father engagement questionnaire. J Child Fam Stud 2018;27:3457–67. 10.1007/s10826-018-1195-0 30369777 PMC6182713

[57] Dorsey S, Kerns SE, Trupin EW, et al Child welfare caseworkers as service brokers for youth in foster care: findings from project focus. Child Maltreat 2012;17:22–31. 10.1177/1077559511429593 22222293

[58] Spradley J The ethnographic interview. Long Grove, IL: Waveland Press, Inc, 1979.

[59] Palinkas LA, Aarons GA, Horwitz S, et al Mixed method designs in implementation research. Adm Policy Ment Health 2011;38:44–53. 10.1007/s10488-010-0314-z 20967495 PMC3025112

[60] Bradley EH, Curry LA, Devers KJ Qualitative data analysis for health services research: developing taxonomy, themes, and theory. Health Serv Res 2007;42:1758–72. 10.1111/j.1475-6773.2006.00684.x 17286625 PMC1955280

[61] Palinkas LA Qualitative and mixed methods in mental health services and implementation research. J Clin Child Adolesc Psychol 2014;43:851–61. 10.1080/15374416.2014.910791 25350675 PMC4212209

[62] Brookman-Frazee L, Stahmer A, Stadnick N, et al Characterizing the use of research-community partnerships in studies of evidence-based interventions in children’s community services. Adm Policy Ment Health 2016;43:93–104. 10.1007/s10488-014-0622-9 25578512 PMC4500757

[63] Curran GM, Bauer M, Mittman B, et al Effectiveness-implementation hybrid designs: combining elements of clinical effectiveness and implementation research to enhance public health impact. Med Care 2012;50:217–26. 10.1097/MLR.0b013e3182408812 22310560 PMC3731143

[64] Leon AC, Davis LL, Kraemer HC The role and interpretation of pilot studies in clinical research. J Psychiatr Res 2011;45:626–9. 10.1016/j.jpsychires.2010.10.008 21035130 PMC3081994

[65] Stokes JO, Jent JF, Weinstein A, et al Does practice make perfect? The relationship between self-reported treatment homework completion and parental skill acquisition and child behaviors. Behav Ther 2016;47:538–49. 10.1016/j.beth.2016.04.004 27423169

[66] Robinson EA, Eyberg SM The dyadic parent-child interaction coding system: standardization and validation. J Consult Clin Psychol 1981;49:245–50. 10.1037/0022-006X.49.2.245 7217491

[67] Boggs SR, Eyberg S, Reynolds LA Concurrent validity of the eyberg child behavior inventory. J Clin Child Psychol 1990;19:75–8. 10.1207/s15374424jccp1901_9

[68] Proctor E, Silmere H, Raghavan R, et al Outcomes for implementation research: conceptual distinctions, measurement challenges, and research agenda. Adm Policy Ment Health 2011;38:65–76. 10.1007/s10488-010-0319-7 20957426 PMC3068522

[69] Weiner BJ, Lewis CC, Stanick C, et al Psychometric assessment of three newly developed implementation outcome measures. Implement Sci 2017;12:108 10.1186/s13012-017-0635-3 28851459 PMC5576104

