# Supplementary material for: Developing and evaluating a lay health worker delivered implementation intervention to decrease engagement disparities in behavioural parent training: a mixed methods study protocol
Source: BMJ Open. 2019 Jul 18;9(7):e028988. doi: 10.1136/bmjopen-2019-028988 (PMC6661633; doi:10.1136/bmjopen-2019-028988)
Supplement: Author's manuscript [file bmjopen-2019-028988.draft_revisions.pdf]

BMJ Open is committed to open peer review. As part of this commitment we make the peer review history of every article we publish publicly available.

When an article is published we post the peer reviewers' comments and the authors' responses online. We also post the versions of the paper that were used during peer review. These are the versions that the peer review comments apply to.

The versions of the paper that follow are the versions that were submitted during the peer review process. They are not the versions of record or the final published versions. They should not be cited or distributed as the published version of this manuscript.

BMJ Open is an open access journal and the full, final, typeset and author-corrected version of record of the manuscript is available on our site with no access controls, subscription charges or pay-per-view fees (http://bmjopen.bmj.com).

If you have any questions on BMJ Open's open peer review process please email info.bmjopen@bmj.com

BMJ Open

# A Mixed Methods Study to Develop and Evaluate a Lay Health Worker Delivered Implementation Intervention to Decrease Engagement Disparities in Behavioral Parent Training

| | |
|---|---|
| Journal: | *BMJ Open* |
| Manuscript ID | bmjopen-2019-028988 |
| Article Type: | Protocol |
| Date Submitted by the Author: | 07-Jan-2019 |
| Complete List of Authors: | Barnett, Miya; University of California Santa Barbara, Counseling, Clinical, and School Psychology<br>Miranda, Jeanne; University of California, Los Angeles , Department of Psychiatry and Biobehavioral Sciences<br>Kia-Keating, Maryam; University of California Santa Barbara, Counseling, Clinical, and School Psychology<br>Saldana, Lisa; Oregon Social Learning Center,<br>Landsverk, John ; Oregon Social Learning Center,<br>Lau, Anna; University of California, Los Angeles, Psychology |
| Keywords: | Lay Health Workers, Behavioral Parent Training, Mental Health Disparities, Implementation Strategies |
| | |

SCHOLARONE™
Manuscripts

# A Mixed Methods Study to Develop and Evaluate a Lay Health Worker Delivered Implementation Intervention to Decrease Engagement Disparities in Behavioral Parent Training

Miya L. Barnett[1] mbarnett@ucsb.edu

Jeanne Miranda[2] jmmiranda@mednet.ucla.edu

Maryam Kia-Keating[1] maryamkk@ucsb.edu

Lisa Saldana[3] lisas@oslc.org

John Landsverk[3] jlandsverk@aol.com

Anna S. Lau[4] alau@psych.ucla.edu

[1] University of California, Santa Barbara, Department of Counseling, Clinical, & School Psychology, Santa Barbara, 93106-9490

[2] University of California, Los Angeles, Department of Psychiatry and Biobehavioral Sciences, Los Angeles, 90095

[3] Oregon Social Learning Center, 10 Shelton McMurphey Blvd, Eugene, OR 97401

[4] University of California, Los Angeles, Department of Psychology, Los Angeles, 90095

Corresponding Author:

Miya Barnett
Assistant Professor
University of California, Santa Barbara
Department of Counseling, Clinical, and School Psychology
Gevirtz Graduate School of Education
Santa Barbara, CA 93106-9490
mbarnett@ucsb.edu
805-893-7459

Word Count: 3,995

For peer review only

## Abstract

**Introduction:** Behavioral parent training programs (BPTs) are effective in preventing and treating early-onset conduct problems and child maltreatment. Unfortunately, pervasive mental health service disparities continue to limit access to and engagement in these interventions. Furthermore, challenges with parental engagement can impede the successful implementation of evidence-based practices (EBPs) in community settings that serve low-income, ethnic minority families. Lay health workers (LHWs)-- individuals without formal mental health training-- represent an important workforce to increase engagement, as they are members of the communities they serve. However, the mobilization of LHWs has not been well studied as an implementation strategy to extend the reach or effectiveness of EBPs in the United States. LHW-delivered implementation interventions that specifically support the engagement of Latinx parents in evidence-based BPTs have the potential to improve clinical and implementation outcomes.

**Methods and Analysis:** A community-partnered approach will use the Quality Implementation Framework[1] to tailor and implement a LHW-delivered implementation intervention that aims to promote Latinx parent engagement in BPTs. Steps from the QIF will guide study activities to: 1) conduct a mixed methods needs assessment to fit the implementation intervention to the local context, 2) adapt LHW delivered implementation strategies to promote parent access to and engagement in Parent-Child Interaction Therapy, and 3) conduct a hybrid effectiveness-implementation pilot trial to examine the feasibility, acceptability, and preliminary effectiveness of the LHW implementation intervention at increasing engagement.

**Ethics and dissemination:** Study procedures have been approved by institutional review board (IRB) at the University of California, Santa Barbara. Results will be shared with the community-

advisory group, at community-based meetings for other stakeholders involved in the pilot project, and submitted for publication in peer-reviewed journals.

## Article Summary

### Strengths and Limitations of this Study

- This study seeks to develop and test an implementation intervention to address the impact of underutilization and poor engagement in BPTs, which limit their clinical effectiveness and successful implementation and sustainment.
- This study aims to improve mobilization of LHWs, who may be able to offer cultural and linguistic bridges to reach diverse families, as a potential solution to address racial/ethnic disparities in engagement in BPTs.
- As a pilot, this study is limited in its sample size to determine the effectiveness of the implementation intervention.
- This study will be limited in its generalizability due to the small sample size, the focus on one BPT (Parent-Child Interaction Therapy), and the characteristics of the local context.

**Keywords:** Lay Health Workers, Implementation Strategies, Behavioral Parent Training, Mental Health Disparities

For peer review only

## Introduction

Early onset conduct problems and child maltreatment have been shown to have enormous personal and societal costs, including long-term mental health and substance abuse problems, higher service utilization, and future abuse against women and children.[2–4] Given that behavioral parent training programs (BPTs) have been shown to be effective at preventing and treating both child maltreatment[5] and conduct problems,[6] large systems of care have invested millions of dollars in the implementation of these interventions.[7,8] Even with major implementation efforts, challenges remain with engagement and retention of families in BPTs.[9,10] Less than half of eligible families enroll in BPTs when they are available,[9] and attrition rates can exceed 65% in community settings.[11–13] The consequences of poor participation in BPTs are significant for individuals and service systems. Families who drop out of treatment are less likely to experience improvements in parenting skills or child disruptive behaviors.[14] Moreover, failed efforts to recruit and retain parents are costly for providers.[7,8,15] Frequent cancellations and no-shows leads to fewer billable hours for community agencies, which are often under immense financial pressure.[15,16] Further, when agencies have inadequate referrals for an evidence-based practice (EBP), such as BPTs, therapists may not learn to deliver the practice with fidelity.[7,8,17] In order to meet the public health potential of BPTs, implementation interventions are needed to support parental engagement. Implementation interventions, which are usually complex and multilevel, include strategies to enhance the adoption and ongoing implementation of clinical interventions at the organization, provider, and consumer levels.[18]

### Addressing Mental Health Service Disparities

Accumulating evidence suggests that BPTs are effective in "real world" settings [19] and with Latinx, African American, and Asian families.[20–22] However, significant mental health

For peer review only

service disparities have been documented for racial and ethnic minority children.[23–25] For example, African American and Latinx children are almost 50% less likely than non-Latinx, White children to receive mental health services for externalizing disorders.[24] Negative attitudes, social norms about mental health treatment, and structural barriers to care (e.g., lack of transportation) all influence help-seeking amongst underserved parents.[26, 27] Beyond challenges that impact the demand for BPTs, systemic barriers limit the supply of these EBPs. Organizational factors that impact service delivery include challenges recruiting and retaining staff that can provide culturally and linguistically appropriate services. Further, mental health professionals often have competing demands on their time beyond their clinical services (e.g., billing, outreach), which limits the number of families they are able to serve.[28] As such, implementation interventions may need to be developed to improve engagement in BPTs for ethnic minority parents to achieve improved engagement.

Powell and colleagues[29] have identified multiple implementation strategies, which focus on increasing consumer engagement with EBPs, including: 1) increasing demand for EBPs, 2) intervening with consumers to enhance uptake and adherence, and 3) preparing consumers to be active participants in their treatment. These implementation strategies are consistent with evidence-based approaches to improve engagement in children's mental health care, which include assessment of barriers, accessibility promotion (e.g., providing child-care or services in the home), psychoeducation about services, and appointment reminders.[30,31] However, limited research has examined whether and how these consumer-facing strategies might increase the success of EBP implementation.

Lay Health Workers (LHWs) have been identified as an important paraprofessional workforce to address service disparities for underserved, low-resource communities.[32] LHWs

For peer review only

(which encompass a range of other titles including: *promotores*, community health workers, peer support partners, wellness navigators, and natural helpers) are individuals without formal mental health training, who have roles intended to increase their community's access to and benefit from services.[33,34] LHWs have the potential to address both demand and supply drivers of disparities in EBP delivery, as illustrated in Figure 1.[32] Demand for EBPs, or the willingness to access services and persist in care, is impacted by an individual's mental health literacy, stigma towards mental illness and help seeking, perceptions of treatment providers, and culturally based beliefs and preferences.[28,35] Systemic barriers to care may exacerbate disparities in seeking and accessing care. For example, undocumented immigrants are especially unlikely to seek mental health services due to fear of being reported to authorities and being discriminated against.[36] Since LHWs come from similar cultural and personal backgrounds as the individuals they serve, they may be especially adept at helping patients overcome distrust of health systems.[37] Regarding supply, the number of professional mental health providers who can deliver linguistically and culturally competent EBPs is inadequate.[38] Furthermore, bilingual, bicultural mental health professionals are frequently tasked with time-consuming demands that extend beyond providing psychotherapeutic interventions, to promote EBP engagement for their underserved communities, such as conducting outreach and case management.[17] The time spent on these tasks might limit the number of individuals they are able to serve. If LHWs are trained to deliver these auxiliary services, it could reduce the burden on mental health professionals and allow them to focus on activities that require advanced training and licensure, such as direct mental health services.[39] However, limited research has focused on how LHW-delivered strategies impact implementation and clinical outcomes of EBPs and if they successfully reduce disparities in engagement. Furthermore, research on LHWs has not consistently reported the

For peer review only

implementation supports (e.g., training and consultation) that are necessary for this non-professional workforce to deliver or support EBPs.[32,34]

The majority of mental health research with LHWs has been conducted in low- and middle-income countries, with emerging evidence that LHWs can improve mental health outcomes when they are tasked with delivering EBPs.[34,40,41] Given that LHWs have effectively addressed mental health disparities globally, it is imperative that this knowledge is translated to increase parity in mental health care for underserved children and families in the United States. LHWs likely will fulfill different roles in high-income countries as compared to low- and middle-income countries where LHWs typically deliver the EBPs using a task-shifting model.[34],[40] Domestically, licensure and certification requirements frequently restrict EBP delivery to mental health professionals, requiring LHWs to have complementary and distinct roles within the provision of EBPs.[10,32] As trusted members of the community, LHW are particularly well suited to deliver implementation strategies that focus on promoting parent entry into and engagement in care (e.g., outreach and adherence promotion).[29,31,32] LHW models of care delivery show great potential to address mental health disparities domestically for underserved children and families; however, significant gaps in the literature limit our understanding of how to effectively mobilize LHW within community mental health services. The proposed study seeks to address these gaps through the development and evaluation of LHWs Enhancing Engagement for Parents (LEEP), an implementation intervention to improve engagement for low-income, Latinx parents into one BPT, Parent-Child Interaction Therapy (PCIT).[42]

**Parent-Child Interaction Therapy**

For peer review only

PCIT has unique benefits and challenges related to engaging parents in treatment. The treatment model uses in vivo feedback (i.e., coaching) to overcome challenges that are inherent to teaching methods used in other BPTs (e.g., didactics, discussion) as it necessitates active participation and assesses learning in real time. PCIT requires that parents demonstrate a high-level of proficiency with the targeted parenting skills before they advance from the first phase of treatment, which focuses on enhancing the parent-child relationship, to the second phase of treatment, which focuses on effective and developmentally appropriate limit setting and discipline approaches, and then until they graduate from treatment.[42] Mastery-based criteria guarantees that all parents can successfully use the skills by the time they complete treatment; however, parents often drop out before they learn limit-setting and discipline skills,[11] which are necessary for long-term decreases in disruptive behaviors.[14] Furthermore, some research suggests that low-income, ethnic and racial minority parents require more practice and time in treatment to reach this level of skill proficiency.[20],[43],[44] Problems with family attendance, retention, and prolonged skill acquisition have downstream effects on PCIT provider implementation outcomes in systems of care. It can take up to three years for mental health professionals to meet PCIT certification requirements (i.e., achieving fidelity and graduating two cases).[45] Thus, low parental engagement results in provider attrition from training, which in turn compromises the sustainability of the intervention, and limits the return on costly investments made to implement PCIT in public service systems.[7,8]

LEEP seeks to improve the supply of and demand for PCIT in agencies that predominately serve low-income, Latinx immigrant families, and address engagement challenges that impact clinical and implementation outcomes (Figure 1). LEEP will be compared to PCIT implementation-as-usual to see if parental engagement and implementation challenges are

For peer review only

ameliorated. A community-partnered approach will focus on making LEEP a feasible and acceptable implementation intervention, with the following three aims:

**Aim 1:** Assess the current context of LHW mobilization in children's mental health services, to inform an implementation intervention (i.e., LEEP) focused on improving parental service entry and engagement in BPT.

**Aim 2:** Through community-partnership, develop a structure for the implementation of LEEP in publicly-funded, children's mental health settings.

**Aim 3:** Evaluate the feasibility of implementing LEEP in community mental health agencies through a pilot effectiveness-implementation trial.

## Methods and Analysis

### Conceptual Framework and Approach

The Quality Implementation Framework (QIF), which includes four phases to support high quality implementation, informs the three study aims and plans for scaling-up LEEP (Figure 2).[1] The first phase of the QIF focuses on various assessment strategies related to organizational needs, innovation-organizational fit, and a capacity or readiness assessment. In Aim 1, these assessments will be conducted through survey data and stakeholder interviews. Phase 2 in the QIF focuses on the development of implementation structures, which will occur during the second aim of this study through the input of a community-advisory group. The community-advisory group will collaboratively help to develop an implementation plan delineating tasks and timelines to establish infrastructure for LHW capacity building, including job descriptions, training plans, and measurement development. Phase 3 of the QIF includes three main activities that will take place during a hybrid type 2 effectiveness-implementation pilot stepped-wedge trial of LEEP. These activities include (1) providing implementation support strategies (e.g.,

For peer review only

supervision and consultation) to LHWs, (2) conducting a process evaluation to identify successes and barriers to implementation (e.g., implementation costs, number of families enrolled in PCIT), and (3) providing ongoing feedback to organizations about the impact LEEP is having on service outcomes. Finally Aim 3 activities will inform Phase 4 of the QIF, which focuses on learning from initial implementation experiences to inform future efforts to scale-up and sustain LEEP.

**Aim 1:** Assess the current context of LHW mobilization in children's mental health services, to inform the implementation of the LEEP model.

**Participants**. Surveys will be administered to LHWs employed or contracted by children's mental health agencies in two counties in California. Approximately 70 to 100 LHWs will be recruited to complete the quantitative survey. Based on a national survey of LHW[46] and California's demographics, LHW are expected to be Latinx, female, and have below a college level of education. Ten agency community mental health agency leaders and 25-30 LHW will be invited to participate in hour-long interviews to expand on the findings from the survey. As LHWs may be monolingual Spanish speakers, survey and interviews will be offered in Spanish or English.

**Procedure.** A mixed-method needs assessment will be conducted to understand how LHWs are currently mobilized in children's community mental health settings, with the purpose of adapting LEEP to fit within the local context. Surveys will provide a breadth of information and qualitative interviews will provide depth of information, to understand perceived barriers to parental engagement in children's mental health services, LHW roles and integration into services, and LHW knowledge about and attitudes towards BPTs and evidence-based engagement strategies.

For peer review only

**Survey measures**. Surveys will be collected via electronic or paper-and-pencil survey based on LHW preferences.

***LHW characteristics***.[47] A demographic questionnaire will provide information about the LHWs personal characteristics, including gender, race/ethnicity, country of origin, educational level, and years of experience as an LHW.

***Cultural background questionnaire.***[48] The Cultural Background Questionnaire is a 19-item self-report measure used to assess therapist generational status and acculturation, including cultural identity (i.e., U.S. identity and Heritage Cultural Identity) and language use.

***Parental engagement.***[49,50] A questionnaire that was developed to measure provider's perceptions of and strategies for engaging fathers has been adapted to measure LHW's perceptions of barriers to engagement, strategies for engagement, and confidence in engagement for parents. Instead of solely investigating father engagement, the questionnaire included perceived barriers in engagement for parents in general, and the LHW's use of and confidence with engagement strategies for both mothers and fathers.

***Attitudes towards BPT strategies.*** A four-item questionnaire was developed for this study to measure LHWs attitudes towards (e.g., *"It is important to teach parents how to…"*) teaching parents common strategies targeted in BPTs including play to improve the parent-child relationship, praise of positive behaviors, ignoring minor misbehaviors, and time-out as a form of discipline.

***EBP questionnaire***.[51] A questionnaire developed to measure service broker's knowledge of and referrals to EBPs has been adapted to identify if LHWs are aware of, making referrals to, and supporting families involved specifically in PCIT(e.g., *"Have you referred parents to Parent-Child Interaction Therapy (PCIT)").*

For peer review only

**Semi-Structured Interviews.** Interview guides will include topics, questions, and probes related to LHW roles, training needs, and experiences and attitudes related to BPTs. Questions will investigate how LHWs became involved in mental health or family support services, descriptions of their job responsibilities, their perceptions of parenting practices targeted in BPTs, their formal and informal preparation for their position, and their training needs. Interviews with agency leaders will focus on how LHW are integrated into services, LHW training, outreach and engagement strategies and challenges with Latinx families, and financing strategies for LHW. A "funnel-approach" will be used with broad-open ended questions related to roles, trainings, and attitudes asked first, followed with specific probes to elicit details.[52]

**Analysis.** A QUAN + QUAL mixed methods design will be used, with quantitative and qualitative data collected simultaneously and given equal weight in analyses. Combined analyses will be used for *complementarity*, with the surveys providing a breadth of information and the interviews providing depth of understanding. Using recommendations for mixed-method design in implementation science, datasets will be merged with qualitative responses included next to quantitative ratings (i.e., triangulation) to analyze if the qualitative and quantitative data provide the same answer to the same question (i.e., convergence), and qualitative data will provide understanding to unexpected quantitative findings (i.e., expansion).[53] Interviews will be transcribed and entered into *NVivo*, software that aids the coding, organization, and retrieval of codes. An iterative process will be used where the coding team first develops a preliminary coding scheme and applies it to a sample text to ensure all relevant themes are captured. Once a final coding scheme is decided upon, coders will apply the final code list to all transcripts. Regular meetings with the coding team will be conducted to examine coding across analysts, resolve differences in coding, conduct iterative refinement of code definitions and the logic of

For peer review only

the coding tree, and collaborate on the development of themes. Qualitative themes will be identified through analysis of co-occurring codes and text analysis.[54],[55]

**Aim 2:** Through community-partnership, develop a structure for the implementation of LEEP in publicly-funded, children's mental health settings.

**Participants.** A community-advisory group with 6-9 stakeholders will be formed to make sure that implementation supports match the unique local context. Agency leaders, PCIT therapists, and LHWs will be represented in the advisory group. Given the wide diversity of viewpoints, education levels, and ethnicities, efforts will be made to provide each participant with equal representation, opportunities for contribution, and honorariums.

**Procedure.** In line with the Model of Research-Community Partnership, which was specifically developed for research in children's mental health services, the formation of the partnership will focus on building relationships, trust, establishing a joint mission, and identifying roles and responsibilities of different partner members. This will provide the foundation to build a synergistic, collaborative relationship focused on developing and delivering LEEP, which in turn could improve the successful and sustained implementation of PCIT.[56] Using data from Aim 1 and in collaboration with the community-advisory group, the LEEP implementation intervention will be adapted from an existing protocol focused on LHW-delivered parent outreach and engagement strategies. This protocol was developed to increase access to PCIT in a low-income, Latinx community in the Southeastern United States, but has not been disseminated to other communities.[47] The implementation supports needed for LHWs to deliver LEEP also will be identified and put into place. Steps from the QIF will be used to guide the activities of the community-advisory group in adapting these materials and developing LEEP's implementation structure.[1] Advisory group meetings will include: 1) trust building

activities, 2) developing a shared mission statement, 3) providing feedback on adaptations needed for LEEP materials, 4) advisory group providing feedback and additional interpretation of survey and interview results from Aim 1, 5) development of an implementation plan that has specific tasks, roles, and a timeline for LEEP implementation, and 6) developing tracking systems to monitor LEEP implementation.

**Aim 3:** Examine the feasibility of LEEP on LHW, clients, and system level targets and outcomes.

In Aim 3, an effectiveness/ implementation hybrid design (Type 2) pilot study will integrate qualitative and quantitative data to examine the feasibility of delivering and scaling-up LEEP. Type 2 hybrid trials simultaneously measure the clinical effectiveness of an intervention, in this case PCIT, and the feasibility and utility of an implementation intervention, which is LEEP.[57] Pilot studies are limited in their ability to test effectiveness given small sample sizes, but they provide a critical phase of research design that can examine the feasibility of the approach to be used in a large-scale study.[58] A focus will be placed on measuring engagement outcomes at a client- and agency-levels to evaluate if LEEP is increasing the reach of PCIT services, service entry, and family treatment engagement.

**Procedure.** Three agency sites that provide PCIT will be involved in this pilot study, which will use a stepped wedge design. In a stepped wedge design, a period of baseline measurement will occur for all sites, in which PCIT will be implemented-as-usual. Then at subsequent time points each site will be randomized to LEEP and response to the intervention will be measured for client and implementation outcomes (Figure 3). At each agency, one to two LHWs (4-6 total) will be trained to deliver LEEP. Client and implementation outcomes will be collected during PCIT implementation-as-usual and LEEP implementation.

For peer review only

**Participants.** Four to six LHWs will be trained to provide LEEP. These LHWs will provide LEEP care extension services to approximately PCIT clients each (16-24 families). LHWs will be trained to provide informed consent for parents. Families that participate in LEEP or PCIT implementation-as-usual will meet criteria for receiving this BPT. This includes having a child between the ages of 2 to 7 and presenting problems consistent with disruptive behaviors, or risk for child maltreatment.

**LEEP Intervention**. The LEEP intervention will include components for LHWs to increase referrals for families to treatment, address barriers to care, and support parents use of skills taught in PCIT within community settings. LHWs will conduct community presentations about PCIT in locations with parents of young children (e.g., Head Start Centers) and health fairs to increase service entry. LHWs will be provided with electronic tablets with e-books that included scripts and videos to use in one-on-one meetings with parents before they enter into care and while they are receiving PCIT services. The e-books will have materials to help LHWs promote enrollment (e.g., parent testimonials), homework adherence, and skill practice (e.g., video demonstrations of the targeted parenting skills).

**Effectiveness outcome measures.** Given that PCIT is an assessment-driven BPT, clinical outcomes will be assessed using standard measures that are collected as part of the routine PCIT protocol. Parents will not complete any additional measures for this study.

*Engagement.* To assess if LEEP impacts engagement at the family-level, session attendance, the percentage of days that daily skill was completed, graduation from PCIT, and the number of sessions needed to graduate will be assessed.

*The Dyadic Parent-Child Interaction Coding System (DPICS).*[59] The DPICS is a behavioral observation coding system that was designed to measure the quality of interaction in

For peer review only

parent-child dyads, which has good interrater reliability. This study will use the DPICS categories, *Behavior Description, Labeled Praise, Unlabeled Praise, Reflection, Question, Negative Talk, and Indirect and Direct Commands,* to determine when the parent's skill acquisition.

*Eyberg Child Behavior Inventory (ECBI).* The ECBI (Eyberg & Pincus, 1999) is a 36-item parent-rating scale of disruptive behavior problems for children between the ages of 2 to 16. Parents rate the frequency of each disruptive behavior on a 7-point Likert scale ranging from *never* (1) to *always* (7), which are summed to yield the Intensity Scale and whether this behavior is a problem for them, with the total number of Yes responses yielding the Problem Scale.

**Implementation outcomes.** Using the implementation outcomes outlined by Proctor and colleagues,[60] this study uses mixed methods to understand the acceptability, appropriateness, feasibility, reach, and costs of delivering LEEP.

***LHW-level outcomes.*** To measure changes in LHW knowledge, perceptions of acceptability and feasibility of PCIT, and competence, LHW will complete pre- and post- self-report and behavioral measures (Table 1). Ongoing fidelity monitoring will be conducted throughout LHW's delivery of LEEP. Fidelity monitoring will include reviewing data capture of the ebook created for LEEP, which will include videos about PCIT and scripts for the LHWs to use with the families they serve. LHWs will also use the iPads that house the ebooks to audio record their sessions. Audio recordings will be used to monitor fidelity to the LEEP model, including addressing barriers to home practice, modeling PCIT skills, conducting skills practice with parents, and providing feedback for skills.

For peer review only

***Implementation costs.*** Costs associated with delivering LEEP will be measured by calculating time estimates associated with all aspects of implementation, including LHW training and service delivery.

***Agency efficiencies***. To identify if LEEP impacts agency-efficiencies, with therapists increasing their billable hours, administrative claims will be calculated for PCIT therapists to measure the time spent in direct services.

***Reach and penetration.*** At the agency level, reach of PCIT will be assessed by the number of clients that enroll in and graduate from PCIT. Using administrative claims data, penetration at the agency-level will be calculated as the percentage of children receiving PCIT in out of the number of children who are eligible for this EBP. Furthermore, the percentage of families that successfully complete PCIT out of the families enrolled will be calculated.

***Acceptability and feasibility.*** Qualitative interviews will be conducted with the LHWs, agency leaders, and ten parents total to assess their perceptions of LEEP including perceived acceptability, appropriateness, and feasibility, which are important early implementation outcomes.[60]

**Analysis.** This pilot trial is designed to evaluate the feasibility of implementing LEEP and develop tools to measure its clinical and implementation targets and outcomes. The trial is not powered to assess intervention effects. Analyses will focus on establishing the reliability and validity of measures of clinical engagement and implementation outcomes. Qualitative data will be analyzed using the same methodology described in Aim 1. Qualitative and quantitative data will be given equal weight in analyses with a focus on *convergence*, *expansion*, and *complimentarity,* with quantitative data used to measure outcomes and qualitative data to understand process.[53]

For peer review only

## Discussion

LEEP has the potential for a significant public health impact, by developing an implementation intervention to increase entry and engagement of Latinx parents into BPTs to improve clinical and implementation outcomes. Though LHWs have been identified as an important workforce to address mental health disparities, limited research has evaluated the best strategies to mobilize them to support evidence-based practice implementation in the United States.[34] As a pilot study findings will be limited in power and generalizability. However, the exploratory and development work in this study will provide data on the feasibility and acceptability of LEEP and its preliminary impact on client recruitment, adherence, and retention in PCIT, which will inform future scaling-up of the model.

**Ethics and dissemination:** Study procedures have been approved by the University of California, Santa Barbara IRB. Results will be submitted for publication in peer-reviewed journals. Furthermore, results will be shared with the community-advisory board and other stakeholders involved in the pilot of LEEP.

For peer review only

Table 1. Measures of LHW Training and Outcomes

| **Measure** | **Description** | **Administration** |
|---|---|---|
| *Demographic and Background Form*[47],[48] | Characterizes personal and professional backgrounds of LHW | Aim 1: Survey<br>Aim 3: Training |
| *EBP Questionnaire*[51] | Measures service brokers' awareness of and referrals to EBPs. | Aim 1: Survey |
| *Parent Engagement Strategy Use and Confidence*[49],[50] | Assess use and confidence of engagement strategies with mothers and fathers. | Aim 1: Survey<br>Aim 3: Pre-, Post-Training |
| *Acceptability and Feasibility of PCIT*[61] | Assess LHW perceptions of the acceptability and feasibility of PCIT | Aim 1: Survey<br>Aim 3: Pre-, Post-Training |
| *PCIT Knowledge Quiz* [47] | A quiz that measures the knowledge of PCIT principles and practices. | Aim 3: Pre-, Post-Training |
| *Dyadic Parent-Child Interaction Coding System*[59] | A behavioral observation coding system that assesses parent-child interactions. It will be used to measure LHW's ability to model parenting behaviors. | Aim 3: Pre-, Post-Training |
| *LEEP Fidelity Monitoring* | Review of ebook data capture to see the LEEP resources being used and audio recordings of home sessions. | Aim 3: During consultation |

For peer review only

Figure 1. LEEP's Approach to Address Supply and Demand Determinants of Disparities

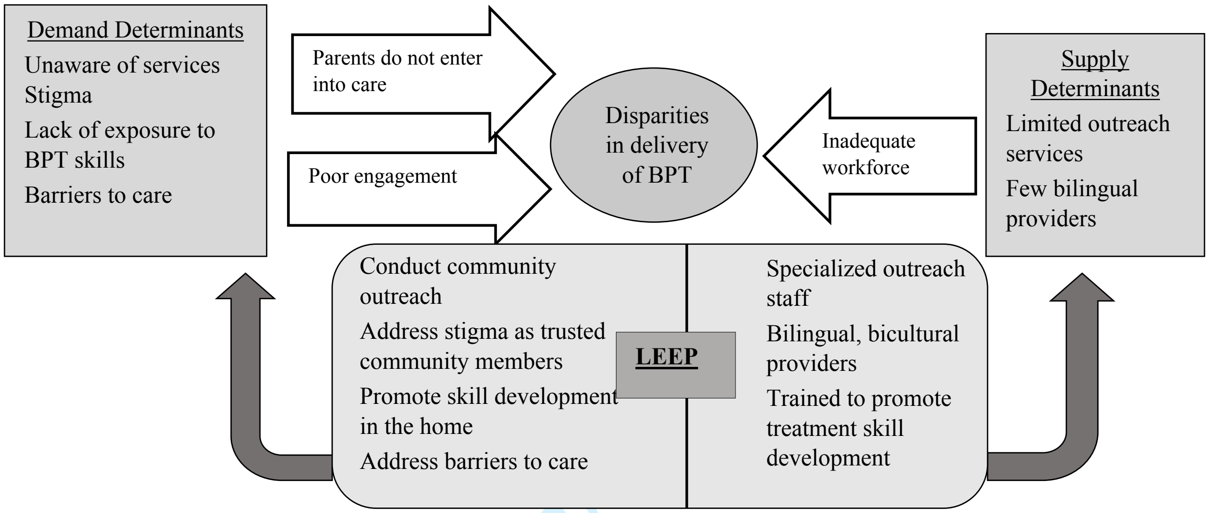


Adapted from Barnett, Lau, & Miranda, 2018. LEEP seeks to address supply and demand drivers of demand for BPTs.

Figure 2. Critical Steps of QIF in LEEP Development and Implementation

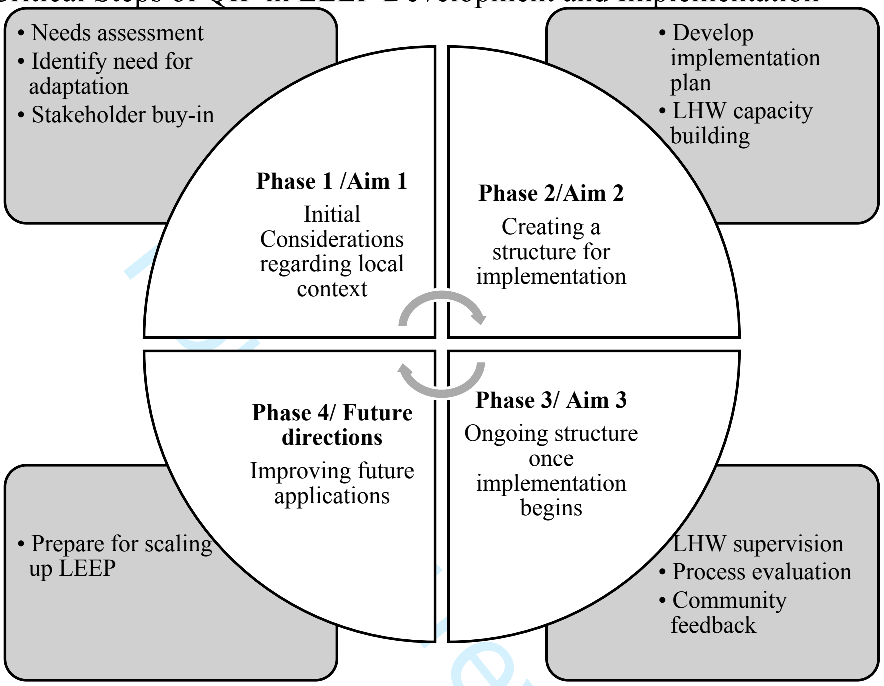


Adapted from Meyers et al. (2012). The steps of the QIF inform each aim of the pilot study and plans for scaling.

Figure 3. Stepped Wedge Trial of LEEP Implementation

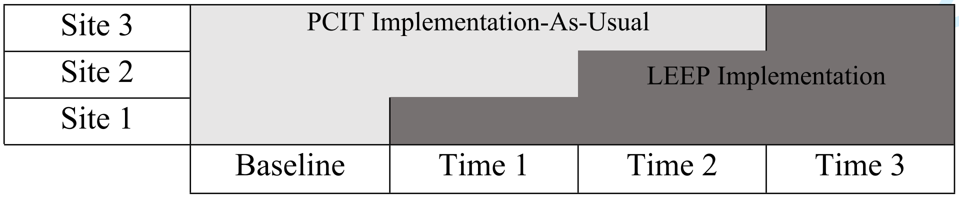


A stepped-wedge design will be used with the three sites implementing LEEP at separate time points.

## References

1. Meyers DC, Durlak JA, Wandersman A. The Quality Implementation Framework: A synthesis of critical steps in the implementation process. Am J Community Psychol. 2012;50(3–4):462–80.

2. Fergusson DM, John Horwood L, Ridder EM. Show me the child at seven: the consequences of conduct problems in childhood for psychosocial functioning in adulthood. J Child Psychol Psychiatry. 2005;46(8):837–49. http://www.ncbi.nlm.nih.gov/pubmed/16033632

3. Rivenbark JG, Odgers CL, Caspi A, Harrington H, Hogan S, Houts RM, et al. The high societal costs of childhood conduct problems: evidence from administrative records up to age 38 in a longitudinal birth cohort. J Child Psychol Psychiatry. 2018;59(6):703–10. http://doi.wiley.com/10.1111/jcpp.12850

4. Prinz RJ, Sanders MR, Shapiro CJ, Whitaker DJ, Lutzker JR. Population-based prevention of child maltreatment: The U.S. Triple P system population trial. Prev Sci. 2016;17(3):410–6.

5. Chen M, Chan KL. Effects of parenting programs on child maltreatment prevention. Trauma, Violence, Abus. 2016;17(1):88–104. http://journals.sagepub.com/doi/10.1177/1524838014566718

6. Kaminski JW, Claussen AH. Evidence base update for psychosocial treatments for disruptive behaviors in children. J Clin Child Adolesc Psychol. 2017;46(4):477–99. https://www.tandfonline.com/doi/full/10.1080/15374416.2017.1310044

7. Beveridge RM, Fowles TR, Masse JJ, McGoron L, Smith MA, Parrish BP, et al. State-wide dissemination and implementation of parent-child interaction therapy (PCIT): Application of theory. Child Youth Serv Rev. 2015;48:38–48. http://dx.doi.org/10.1016/j.childyouth.2014.11.013

8. Timmer SG, Urquiza AJ, Boys DK, Forte LA, Quick-Abdullah D, Chan S, et al. Filling potholes on the implementation highway: Evaluating the implementation of Parent-Child Interaction Therapy in Los Angeles County. Child Abus Negl. 2016;53:40–50. http://dx.doi.org/10.1016/j.chiabu.2015.11.011

9. Baker CN, Arnold DH, Meagher S. Enrollment and attendance in a parent training prevention program for conduct problems. Prev Sci. 2011;12(2):126–38. http://www.springerlink.com/index/J410K861466J4204.pdf

10. Sanders MR. Triple P-Positive Parenting Program as a public health approach to strengthening parenting. J Fam Psychol. 2008;22(4):506–17.

11. Lanier P, Kohl PL, Benz J, Swinger D, Moussette P, Drake B. Parent-child interaction therapy in a community setting: Examining outcomes, attrition, and treatment setting. Res Soc Work Pract. 2011;21(6):689–98. http://journals.sagepub.com/doi/10.1177/1049731511406551

12. Budd KS, Lyon AR. A community mental health implementation of Parent-Child Interaction Therapy (PCIT). J Child Fam Stud. 2010;19(5):654–68.

For peer review only

http://link.springer.com/10.1007/s10826-010-9353-z

13. Pearl E, Thieken L, Olafson E, Boat B, Connelly L, Barnes J, et al. Effectiveness of community dissemination of parent-child interaction therapy. Psychol Trauma Theory, Res Pract Policy. 2012;4(2):204–13. https://bobcat.militaryfamilies.psu.edu/sites/default/files/placed-programs/Pearl et al 2012 effectiveness of community dissemination.pdf

14. Boggs SR, Eyberg SM, Edwards DL, Rayfield A, Jacobs J, Bagner D, et al. Outcomes of parent-child interaction therapy: A comparison of treatment completers and study dropouts one to three years later. Child Fam Behav Ther. 2005;26(4):1–22. http://www.tandfonline.com/action/journalInformation?journalCode=wcfb20

15. Hoagwood KE, Olin SS, Horwitz S, McKay M, Cleek A, Gleacher A, et al. Scaling up evidence-based practices for children and families in New York State: Toward evidence-based policies on implementation for state mental health systems. J Clin Child Adolesc Psychol. 2014;43(2):145–57. http://www.tandfonline.com/doi/abs/10.1080/15374416.2013.869749

16. Stewart RE, Adams DR, Mandell DS, Hadley TR, Evans AC, Rubin R, et al. The perfect storm: Collision of the business of mental health and the implementation of evidence-based practices. Psychiatr Serv. 2016;67(2):159–61. www.ctacny.com/beep-business-

17. Regan J, Lau AS, Barnett M, Stadnick N, Hamilton A, Pesanti K, et al. Agency responses to a system-driven implementation of multiple evidence-based practices in children's mental health services. BMC Health Serv Res. 2017;17(1).

18. Eldh AC, Almost J, Decorby-Watson K, Gifford W, Harvey G, Hasson H, et al. Clinical interventions, implementation interventions, and the potential greyness in between -a discussion paper [Internet]. Vol. 17, BMC Health Services Research. BioMed Central; 2017 [cited 2018 Oct 28]. p. 16. http://www.ncbi.nlm.nih.gov/pubmed/28061856

19. Michelson D, Davenport C, Dretzke J, Barlow J, Day C. Do evidence-based interventions work when tested in the "real world?" A systematic review and meta-analysis of parent management training for the treatment of child disruptive behavior [Internet]. Vol. 16, Clinical Child and Family Psychology Review. 2013 [cited 2018 Sep 22]. p. 18–34. http://www.ncbi.nlm.nih.gov/pubmed/23420407

20. McCabe K, Yeh M. Parent-child interaction therapy for Mexican Americans: A randomized clinical trial. J Clin Child Adolesc Psychol. 2009;38(5):753–9. http://www.ncbi.nlm.nih.gov/pubmed/20183659

21. Ortiz C, Del Vecchio T. Cultural diversity: Do we need a new wake-up call for parent training? Behav Ther. 2013;44(3):443–58. http://www.ncbi.nlm.nih.gov/pubmed/23768671

22. Reid MJ, Webster-stratton C, Beauchaine TP. Parent training in Head Start : A comparison of program response among African American, Asian American, Caucasian, and Hispanic Mothers. Prev Sci. 2002;2(4):209–27. http://link.springer.com/10.1023/A:1013618309070

For peer review only

23. Aratani Y, Cooper JL. Racial and ethnic disparities in the continuation of community-based children's mental health services. J Behav Heal Serv Res. 2012;39(2):116–29. http://www.ncbi.nlm.nih.gov/pubmed/22076314

24. Coker TR, Elliott MN, Kataoka S, Schwebel DC, Mrug S, Grunbaum JA, et al. Racial/ethnic disparities in the mental health care utilization of fifth grade children. Acad Pediatr. 2009;9(2):89–96. https://www.sciencedirect.com/science/article/pii/S1876285908002672

25. Zimmerman FJ. Social and economic determinants of disparities in professional help-seeking for child mental health problems: Evidence from a national sample. Health Serv Res. 2005;40(5 I):1514–33. http://www.ncbi.nlm.nih.gov/pubmed/16174145

26. Lindsey MA, Chambers K, Pohle C, Beall P, Lucksted A. Understanding the behavioral determinants of mental mealth service use by urban, under-resourced black youth: Adolescent and caregiver perspectives. J Child Fam Stud. 2013;22(1):107–21. http://link.springer.com/10.1007/s10826-012-9668-z

27. Gopalan G, Goldstein L, Klingenstein K, Carolyn Sicher Psy D, Blake C, McKay MM. Engaging families into child mental health treatment: Updates and special considerations. J Can Acad Child Adolesc Psychiatry. 2010;19(3):182–96. http://www.ncbi.nlm.nih.gov/pubmed/20842273

28. Kilbourne AM, Switzer G, Hyman K, Crowley-Matoka M, Fine MJ. Advancing health disparities research within the health care system: A conceptual framework. Am J Public Health. 2006;96(12):2113–21. http://www.ncbi.nlm.nih.gov/pubmed/17077411

29. Powell BJ, Waltz TJ, Chinman MJ, Damschroder LJ, Smith JL, Matthieu MM, et al. A refined compilation of implementation strategies: Results from the Expert Recommendations for Implementing Change (ERIC) project. Implement Sci. 2015;10(1):21. http://implementationscience.biomedcentral.com/articles/10.1186/s13012-015-0209-1

30. Becker KD, Lee BR, Daleiden EL, Lindsey M, Brandt NE, Chorpita BF. The common elements of engagement in children's mental health services: Which elements for which outcomes? J Clin Child Adolesc Psychol. 2015;44(1):30–43.

31. Becker KD, Boustani M, Gellatly R, Chorpita BF. Forty years of engagement research in children's mental health services: Multidimensional measurement and practice elements. J Clin Child Adolesc Psychol. 2018;47(1):1–23. https://www.tandfonline.com/doi/full/10.1080/15374416.2017.1326121

32. Barnett ML, Lau AS, Miranda J. Lay health worker involvement in evidence-based treatment delivery: A conceptual model to address disparities in care. Annu Rev Clin Psychol. 2018;14(1):185–208. http://www.annualreviews.org/doi/10.1146/annurev-clinpsy-050817-084825

33. Ayala G, Vaz L, Earp J, Elder J. Outcome effectiveness of the lay health advisor model among Latinos in the United States: an examination by role. Health Educ. 2010; https://academic.oup.com/her/article-abstract/25/5/815/567914

For peer review only

34. Barnett ML, Gonzalez A, Miranda J, Chavira DA, Lau AS. Mobilizing community health workers to address mental health disparities for underserved populations: A systematic review. Adm Policy Ment Heal Ment Heal Serv Res. 2018;45(2):195–211.

35. Alegria M, Vallas M, Pumariega A, Becker KD, Lee BR, Daleiden EL, et al. NIH Public Access. Child Youth Serv Rev. 2015;44(2):181–217. http://www.sciencedirect.com/science/article/pii/S0190740915001085

36. Philbin MM, Flake M, Hatzenbuehler ML, Hirsch JS. State-level immigration and immigrant-focused policies as drivers of Latino health disparities in the United States. Soc Sci Med. 2018;199:29–38. https://www.sciencedirect.com/science/article/pii/S0277953617302307

37. Katigbak C, Van Devanter N, Islam N T-S. Partners in health: A conceptual framework for the role of community health workers in facilitating patients' adoption of healthy behaviors. J Public …. 2015; https://ajph.aphapublications.org/doi/full/10.2105/AJPH.2014.302411

38. Huang L, MacBeth G, Dodge J, Jacobstein D. Transforming the workforce in children's mental health [Internet]. Vol. 32, Administration and Policy in Mental Health. 2004 [cited 2018 Nov 5]. p. 167–87. https://link.springer.com/content/pdf/10.1023/B:APIH.0000042745.64582.72.pdf

39. Barnett ML, Davis EM, Callejas LM, White JV, Acevedo-Polakovich ID, Niec LN, et al. The development and evaluation of a natural helpers' training program to increase the engagement of urban, Latina/o families in parent-child interaction therapy. Child Youth Serv Rev. 2016;65.

40. Singla DR, Kohrt BA, Murray LK, Anand A, Chorpita BF, Patel V. Psychological Treatments for the World: Lessons from Low- and Middle-Income Countries. Annu Rev Clin Psychol. 2017;13(1):149–81. http://www.annualreviews.org/doi/10.1146/annurev-clinpsy-032816-045217

41. Nadja : van Ginneken, Prathap : Tharyan, Simon : Lewin, N : Rao Girish, SM : Meera, Jessica : Pian, et al. Non-specialist health worker interventions for the care of mental, neurological and substance-abuse disorders in low- and middle-income countries SO-: Cochrane Database of Systematic Reviews YR-: 2013 NO-: 11. 2013;(11).

42. Funderburk BW, Eyberg S. Parent–child interaction therapy. In: History of psychotherapy: Continuity and change (2nd ed). Washington: American Psychological Association; p. 415–20. http://content.apa.org/books/12353-021

43. Bernal G. Parent-Child Interaction Therapy for Puerto Rican preschool children with ADHD and behavior Problems : A pilot efficacy study. Fam Process. 2009;48(2):232–52. www.FamilyProcess.org

44. Ramos G, Blizzard AM, Barroso NE, Bagner DM. Parent training and skill acquisition and utilization among Spanish- and English-speaking Latino families. J Child Fam Stud. 2018;27(1):268–79. http://link.springer.com/10.1007/s10826-017-0881-7

45. Scudder AT, Herschell AD. Building an evidence-base for the training of evidence-based

For peer review only

treatments in community settings: Use of an expert-informed approach. Child Youth Serv Rev. 2015;55:84–92. http://dx.doi.org/10.1016/j.childyouth.2015.05.003

46. Ingram M, Reinschmidt KM, Schachter KA, Davidson CL, Sabo SJ, De Zapien JG, et al. Establishing a professional profile of Community Health Workers: Results from a national study of roles, activities and training. J Community Health. 2012;37(2):529–37.

47. Barnett ML, Davis EM, Callejas LM, White J V., Acevedo-Polakovich ID, Niec LN, et al. The development and evaluation of a natural helpers' training program to increase the engagement of urban, Latina/o families in parent-child interaction therapy. Child Youth Serv Rev. 2016;65:17–25.

48. Saifan D, Brookman-Frazee L, Barnett M, Gonzalez JC, Lau AS. Ethnic minority community therapists' acculturation and reported adaptations to children's evidence-based practices. Cult Divers Ethn Minor Psychol. 2018;24(4):530–40.

49. Tully LA, Collins DAJ, Piotrowska PJ, Mairet KS, Hawes DJ, Moul C, et al. Examining practitioner competencies, organizational support and barriers to engaging fathers in parenting interventions. Child Psychiatry Hum Dev. 2018;49(1):109–22. http://www.ncbi.nlm.nih.gov/pubmed/28523378

50. Jiang Y, Tully LA, Burn MT, Piotrowska P, Collins DAJ, Moul C, et al. Development and psychometric evaluation of the father engagement questionnaire. J Child Fam Stud. 2018;27(11):3457–67. http://link.springer.com/10.1007/s10826-018-1195-0

51. Dorsey S, Kerns SEU, Trupin EW, Conover KL, Berliner L. Child welfare caseworkers as service brokers for youth in foster care: Findings from project focus. Child Maltreat. 2012;17(1):22–31.

52. Spradley J. The Ethnographic Interview. The Ethnographic Interview. Long Grove, IL: Waveland Press, Inc.; 1979.

53. Palinkas LA, Aarons GA, Horwitz S, Chamberlain P, Hurlburt M, Landsverk J. Mixed method designs in implementation research. Adm Policy Ment Heal Ment Heal Serv Res. 2011;38(1):44–53.

54. Bradley EH, Curry LA, Devers KJ. Qualitative data analysis for health services research: Developing taxonomy, themes, and theory. Health Serv Res. 2007;42(4):1758–72. http://www.ncbi.nlm.nih.gov/pubmed/17286625

55. Palinkas LA. Qualitative and mixed methods in mental health services and implementation research. J Clin Child Adolesc Psychol. 2014;43(6):851–61. http://www.ncbi.nlm.nih.gov/pubmed/25350675

56. Brookman-Frazee L, Stahmer A, Stadnick N, Chlebowski C, Herschell A, Garland AF. Characterizing the use of research-community partnerships in studies of evidence-based Interventions in children's community services. Adm Policy Ment Heal Ment Heal Serv Res. 2016;43(1):93–104.

57. Curran GM, Bauer M, Mittman B, Pyne JM, Stetler C. Effectiveness-implementation hybrid designs. Med Care. 2012;50(3):217–26. http://www.ncbi.nlm.nih.gov/pubmed/22310560

For peer review only

58. Leon AC, Davis LL, Kraemer HC. Role and interpretation of pilot studies in clinical research. J Psychiatr Res. 2012;45(5):626–9.

59. Robinson EA, Eyberg SM. The dyadic parent–child interaction coding system: Standardization and validation. J Consult Clin Psychol. 1981;49(2):245–50. http://doi.apa.org/getdoi.cfm?doi=10.1037/0022-006X.49.2.245

60. Proctor E, Silmere H, Raghavan R, Hovmand P, Aarons G, Bunger A, et al. Outcomes for implementation research: Conceptual distinctions, measurement challenges, and research agenda. Adm Policy Ment Heal Ment Heal Serv Res. 2011;38(2):65–76. http://www.ncbi.nlm.nih.gov/pubmed/20957426

61. Weiner BJ, Lewis CC, Stanick C, Powell BJ, Dorsey CN, Clary AS, et al. Psychometric assessment of three newly developed implementation outcome measures. Implement Sci. 2017;12(1):108. http://implementationscience.biomedcentral.com/articles/10.1186/s13012-017-0635-3

For peer review only

### Author Statement

MLB is the principal investigator for the study protocol. MLB generated the idea and design of the study, and was the primary writer of the manuscript. ASL, JM, JL, MKK, and LS also made substantial contributions to study conception and design as mentors to MLB for this K01 award. All authors reviewed and provided feedback for this manuscript. The final version of this manuscript was vetted and approved by all authors.

### Funding

This study was funded by the National Institute of Mental Health (K01MH110608) awarded to MLB. Additionally, the preparation of this article was supported in part by the Implementation Research Institute (IRI) at the Brown School, Washington University in St. Louis, through an award from the National Institute of Mental Health (R25 MH080916). MLB is a fellow of IRI.

### Competing interests

None declared

### Ethics Approval

University of California, Santa Barbara IRB approved study procedures (Protocol number: 1-18-0919).

### Acknowledgements

The authors would like to thank Camille Nebeker and Emily Winslow, who serve as consultants on this project. We are grateful to the help provided by Juan Carlos Gonzalez, Corinna Klein, Erika Luis Sanchez, Iliana Flores, and Ana Romero, who assisted on this project. Finally, we would like to thank our community partners at the Santa Barbara Promotora Network and CALM (Child Abuse Mediation and Listening).

For peer review only

# Reporting checklist for protocol of a clinical trial.

Based on the SPIRIT guidelines.

## Instructions to authors

Complete this checklist by entering the page numbers from your manuscript where readers will find each of the items listed below.

Your article may not currently address all the items on the checklist. Please modify your text to include the missing information. If you are certain that an item does not apply, please write "n/a" and provide a short explanation.

Upload your completed checklist as an extra file when you submit to a journal.

In your methods section, say that you used the SPIRIT reporting guidelines, and cite them as:

Chan A-W, Tetzlaff JM, Altman DG, Laupacis A, Gøtzsche PC, Krleža-Jerić K, Hróbjartsson A, Mann H, Dickersin K, Berlin J, Doré C, Parulekar W, Summerskill W, Groves T, Schulz K, Sox H, Rockhold FW, Rennie D, Moher D. SPIRIT 2013 Statement: Defining standard protocol items for clinical trials. Ann Intern Med. 2013;158(3):200-207

| | | Reporting Item | Page Number |
|---|---|---|---|
| Title | #1 | Descriptive title identifying the study design, population, interventions, and, if applicable, trial acronym | 1 |
| Trial registration | #2a | Trial identifier and registry name. If not yet registered, name of intended registry | N/A |
| Trial registration: data set | #2b | All items from the World Health Organization Trial Registration Data Set | N/A |
| Protocol version | #3 | Date and version identifier | N/A |
| Funding | #4 | Sources and types of financial, material, and other support | 28 |
| Roles and responsibilities: contributorship | #5a | Names, affiliations, and roles of protocol contributors | 28 |
| Roles and responsibilities: | #5b | Name and contact information for the trial sponsor | 1 |

For peer review only

| | | | |
|---|---|---|---|
| sponsor contact information | | | |
| Roles and responsibilities: sponsor and funder | #5c | Role of study sponsor and funders, if any, in study design; collection, management, analysis, and interpretation of data; writing of the report; and the decision to submit the report for publication, including whether they will have ultimate authority over any of these activities | 28 |
| Roles and responsibilities: committees | #5d | Composition, roles, and responsibilities of the coordinating centre, steering committee, endpoint adjudication committee, data management team, and other individuals or groups overseeing the trial, if applicable (see Item 21a for data monitoring committee) | 13 |
| Background and rationale | #6a | Description of research question and justification for undertaking the trial, including summary of relevant studies (published and unpublished) examining benefits and harms for each intervention | 4-9 |
| Background and rationale: choice of comparators | #6b | Explanation for choice of comparators | 9 |
| Objectives | #7 | Specific objectives or hypotheses | 9 |
| Trial design | #8 | Description of trial design including type of trial (eg, parallel group, crossover, factorial, single group), allocation ratio, and framework (eg, superiority, equivalence, non-inferiority, exploratory) | 10 |
| Study setting | #9 | Description of study settings (eg, community clinic, academic hospital) and list of countries where data will be collected. Reference to where list of study sites can be obtained | 10 |
| Eligibility criteria | #10 | Inclusion and exclusion criteria for participants. If applicable, eligibility criteria for study centres and individuals who will perform the interventions (eg, surgeons, psychotherapists) | 15 |
| Interventions: description | #11a | Interventions for each group with sufficient detail to allow replication, including how and when they will be administered | 15 |
| Interventions: modifications | #11b | Criteria for discontinuing or modifying allocated interventions for a given trial participant (eg, drug dose change in response to harms, participant request, or improving / worsening disease) | N/A |
| Interventions: adherance | #11c | Strategies to improve adherence to intervention protocols, and any procedures for monitoring adherence (eg, drug tablet return; | 16 |

For peer review only

| | | | |
|---|---|---|---|
| | | laboratory tests) | |
| Interventions: concomitant care | #11d | Relevant concomitant care and interventions that are permitted or prohibited during the trial | N/A |
| Outcomes | #12 | Primary, secondary, and other outcomes, including the specific measurement variable (eg, systolic blood pressure), analysis metric (eg, change from baseline, final value, time to event), method of aggregation (eg, median, proportion), and time point for each outcome. Explanation of the clinical relevance of chosen efficacy and harm outcomes is strongly recommended | 16 |
| Participant timeline | #13 | Time schedule of enrolment, interventions (including any run-ins and washouts), assessments, and visits for participants. A schematic diagram is highly recommended (see Figure) | 15 |
| Sample size | #14 | Estimated number of participants needed to achieve study objectives and how it was determined, including clinical and statistical assumptions supporting any sample size calculations | 15 |
| Recruitment | #15 | Strategies for achieving adequate participant enrolment to reach target sample size | 15 |
| Allocation: sequence generation | #16a | Method of generating the allocation sequence (eg, computer-generated random numbers), and list of any factors for stratification. To reduce predictability of a random sequence, details of any planned restriction (eg, blocking) should be provided in a separate document that is unavailable to those who enrol participants or assign interventions | N/A |
| Allocation concealment mechanism | #16b | Mechanism of implementing the allocation sequence (eg, central telephone; sequentially numbered, opaque, sealed envelopes), describing any steps to conceal the sequence until interventions are assigned | N/A |
| Allocation: implementation | #16c | Who will generate the allocation sequence, who will enrol participants, and who will assign participants to interventions | N/A |
| Blinding (masking) | #17a | Who will be blinded after assignment to interventions (eg, trial participants, care providers, outcome assessors, data analysts), and how | N/A |
| Blinding (masking): emergency | #17b | If blinded, circumstances under which unblinding is permissible, and procedure for revealing a participant's allocated intervention during | N/A |

For peer review only

| | | | |
|---|---|---|---|
| unblinding | | the trial | |
| Data collection plan | #18a | Plans for assessment and collection of outcome, baseline, and other trial data, including any related processes to promote data quality (eg, duplicate measurements, training of assessors) and a description of study instruments (eg, questionnaires, laboratory tests) along with their reliability and validity, if known. Reference to where data collection forms can be found, if not in the protocol | 16 |
| Data collection plan: retention | #18b | Plans to promote participant retention and complete follow-up, including list of any outcome data to be collected for participants who discontinue or deviate from intervention protocols | N/A |
| Data management | #19 | Plans for data entry, coding, security, and storage, including any related processes to promote data quality (eg, double data entry; range checks for data values). Reference to where details of data management procedures can be found, if not in the protocol | N/A |
| Statistics: outcomes | #20a | Statistical methods for analysing primary and secondary outcomes. Reference to where other details of the statistical analysis plan can be found, if not in the protocol | 17 |
| Statistics: additional analyses | #20b | Methods for any additional analyses (eg, subgroup and adjusted analyses) | N/A |
| Statistics: analysis population and missing data | #20c | Definition of analysis population relating to protocol non-adherence (eg, as randomised analysis), and any statistical methods to handle missing data (eg, multiple imputation) | N/A |
| Data monitoring: formal committee | #21a | Composition of data monitoring committee (DMC); summary of its role and reporting structure; statement of whether it is independent from the sponsor and competing interests; and reference to where further details about its charter can be found, if not in the protocol. Alternatively, an explanation of why a DMC is not needed | N/A |
| Data monitoring: interim analysis | #21b | Description of any interim analyses and stopping guidelines, including who will have access to these interim results and make the final decision to terminate the trial | N/A |
| Harms | #22 | Plans for collecting, assessing, reporting, and managing solicited and spontaneously reported adverse events and other unintended effects of trial interventions or trial conduct | N/A |
| Auditing | #23 | Frequency and procedures for auditing trial conduct, if any, and | N/A |

For peer review only

| | | | |
|---|---|---|---|
| | | whether the process will be independent from investigators and the sponsor | |
| Research ethics approval | #24 | Plans for seeking research ethics committee / institutional review board (REC / IRB) approval | 18 |
| Protocol amendments | #25 | Plans for communicating important protocol modifications (eg, changes to eligibility criteria, outcomes, analyses) to relevant parties (eg, investigators, REC / IRBs, trial participants, trial registries, journals, regulators) | N/A |
| Consent or assent | #26a | Who will obtain informed consent or assent from potential trial participants or authorised surrogates, and how (see Item 32) | 15 |
| Consent or assent: ancillary studies | #26b | Additional consent provisions for collection and use of participant data and biological specimens in ancillary studies, if applicable | N/A |
| Confidentiality | #27 | How personal information about potential and enrolled participants will be collected, shared, and maintained in order to protect confidentiality before, during, and after the trial | N/A |
| Declaration of interests | #28 | Financial and other competing interests for principal investigators for the overall trial and each study site | 28 |
| Data access | #29 | Statement of who will have access to the final trial dataset, and disclosure of contractual agreements that limit such access for investigators | N/A |
| Ancillary and post trial care | #30 | Provisions, if any, for ancillary and post-trial care, and for compensation to those who suffer harm from trial participation | N/A |
| Dissemination policy: trial results | #31a | Plans for investigators and sponsor to communicate trial results to participants, healthcare professionals, the public, and other relevant groups (eg, via publication, reporting in results databases, or other data sharing arrangements), including any publication restrictions | 18 |
| Dissemination policy: authorship | #31b | Authorship eligibility guidelines and any intended use of professional writers | 28 |
| Dissemination policy: reproducible research | #31c | Plans, if any, for granting public access to the full protocol, participant-level dataset, and statistical code | N/A |
| Informed consent materials | #32 | Model consent form and other related documentation given to participants and authorised surrogates | N/A |

For peer review only

| | | | |
|---|---|---|---|
| Biological specimens | #33 | Plans for collection, laboratory evaluation, and storage of biological specimens for genetic or molecular analysis in the current trial and for future use in ancillary studies, if applicable | N/A |

The SPIRIT checklist is distributed under the terms of the Creative Commons Attribution License CC-BY-ND 3.0. This checklist was completed on 07. January 2019 using https://www.goodreports.org/, a tool made by the EQUATOR Network in collaboration with Penelope.ai

For peer review only

BMJ Open

# Developing and Evaluating a Lay Health Worker Delivered Implementation Intervention to Decrease Engagement Disparities in Behavioral Parent Training: A Mixed Methods Study Protocol

| | |
|---|---|
| Journal: | *BMJ Open* |
| Manuscript ID | bmjopen-2019-028988.R1 |
| Article Type: | Protocol |
| Date Submitted by the Author: | 22-Mar-2019 |
| Complete List of Authors: | Barnett, Miya; University of California Santa Barbara, Counseling, Clinical, and School Psychology<br>Miranda, Jeanne; University of California Los Angeles<br>Kia-Keating, Maryam; University of California Santa Barbara, Counseling, Clinical, and School Psychology<br>Saldana, Lisa; Oregon Social Learning Center,<br>Landsverk, John ; Oregon Social Learning Center,<br>Lau, Anna; University of California Los Angeles |
| <b>Primary Subject Heading</b>: | Mental health |
| Secondary Subject Heading: | Evidence based practice |
| Keywords: | Lay Health Workers, Behavioral Parent Training, Mental Health Disparities, Implementation Strategies |

SCHOLARONE™
Manuscripts

# Developing and Evaluating a Lay Health Worker Delivered Implementation Intervention to Decrease Engagement Disparities in Behavioral Parent Training: A Mixed Methods Study Protocol

Miya L. Barnett[1] mbarnett@ucsb.edu

Jeanne Miranda[2] jmmiranda@mednet.ucla.edu

Maryam Kia-Keating[1] maryamkk@ucsb.edu

Lisa Saldana[3] lisas@oslc.org

John Landsverk[3] jlandsverk@aol.com

Anna S. Lau[4] alau@psych.ucla.edu

[1] University of California, Santa Barbara, Department of Counseling, Clinical, & School Psychology, Santa Barbara, 93106-9490

[2] University of California, Los Angeles, Department of Psychiatry and Biobehavioral Sciences, Los Angeles, 90095

[3] Oregon Social Learning Center, 10 Shelton McMurphey Blvd, Eugene, OR 97401

[4] University of California, Los Angeles, Department of Psychology, Los Angeles, 90095

Corresponding Author:

Miya Barnett
Assistant Professor
University of California, Santa Barbara
Department of Counseling, Clinical, and School Psychology
Gevirtz Graduate School of Education
Santa Barbara, CA 93106-9490
mbarnett@ucsb.edu
805-893-7459

Word Count: 3995

For peer review only

## Abstract

**Introduction:** Behavioral parent training programs (BPTs) are effective in preventing and treating early-onset conduct problems and child maltreatment. Unfortunately, pervasive mental health service disparities continue to limit access to and engagement in these interventions. Furthermore, challenges with parental engagement can impede the successful implementation of evidence-based practices (EBPs) in community settings that serve low-income, ethnic minority families. Lay health workers (LHWs)-- individuals without formal mental health training-- represent an important workforce to increase engagement, as they are members of the communities they serve. However, the mobilization of LHWs has not been well studied as an implementation strategy to extend the reach or effectiveness of EBPs in the United States. LHW-delivered implementation interventions that specifically support the engagement of Latinx parents in evidence-based BPTs have the potential to improve clinical and implementation outcomes.

**Methods and Analysis:** A community-partnered approach will use the Quality Implementation Framework (QIF) to tailor and implement a LHW-delivered implementation intervention that aims to promote Latinx parent engagement in BPTs. Steps from the QIF will guide study activities to: 1) conduct a mixed methods needs assessment to fit the implementation intervention to the local context, 2) adapt LHW delivered implementation strategies to promote parent access to and engagement in Parent-Child Interaction Therapy, and 3) conduct a hybrid effectiveness-implementation pilot trial to examine the feasibility, acceptability, and preliminary effectiveness of the LHW implementation intervention at increasing engagement.

**Ethics and dissemination:** Study procedures have been approved by institutional review board (IRB) at the University of California, Santa Barbara. Results will be shared with the community-

advisory group, at community-based meetings for other stakeholders involved in the pilot project, and submitted for publication in peer-reviewed journals.

## Article Summary

**Strengths and Limitations of this Study**

- This study seeks to develop and test an implementation intervention to address the impact of underutilization and poor engagement in BPTs, which limit their clinical effectiveness and successful implementation and sustainment.
- This study aims to improve mobilization of LHWs, who may be able to offer cultural and linguistic bridges to reach diverse families, as a potential solution to address racial/ethnic disparities in engagement in BPTs.
- As a pilot, this study is limited in its sample size to determine the effectiveness of the implementation intervention.
- This study will be limited in its generalizability due to the small sample size, the focus on one BPT (Parent-Child Interaction Therapy), and the characteristics of the local context.

**Keywords:** Lay Health Workers, Implementation Strategies, Behavioral Parent Training, Mental Health Disparities

For peer review only

## Introduction

Early onset conduct problems and child maltreatment have been shown to have enormous personal and societal costs, including long-term mental health and substance abuse problems, higher service utilization, and future abuse against women and children.[1–3] Given that behavioral parent training programs (BPTs) have been shown to be effective at preventing and treating both child maltreatment[4] and conduct problems[5] for racially and ethnically diverse families,[6,7] large systems of care have invested millions of dollars in the implementation of these interventions.[8,9] Even with major implementation efforts, challenges remain with engagement and retention of families in BPTs.[10,11] A systematic review of engagement in BPTs found that at least 25% of families that are appropriate for BPTs do not enroll in treatment, and an additional 26% begin, but then drop out of treatment, with higher rates of attrition for low-SES families.[12] In fact, in community implementation of BPTs, attrition rates can exceed 65%.[13–15]

The consequences of poor participation in BPTs are significant. Families who drop out of treatment are less likely to experience improvements in parenting skills or child disruptive behaviors.[16] Moreover, failed efforts to recruit and retain parents are costly for providers.[17] Frequent cancellations and no-shows leads to fewer billable hours for community agencies, which are often under immense financial pressure.[17,18] Further, inadequate referrals negatively impacts the implementation of evidence-based practice (EBP), as therapists may not learn to deliver the practice with fidelity.[8,9,19] Challenges with engagement may be especially pronounced for racial and ethnic minority families, as mental health service disparities have been well documented.[20] For example, African American and Latinx children are almost 50% less likely than non-Latinx, White children to receive treatment for externalizing disorders.[21]

For peer review only

In order to meet the public health potential of BPTs and address service disparities, implementation interventions are needed to support parental engagement for ethnic minority parents. Implementation interventions, which are usually complex and multilevel, include strategies to enhance the adoption and ongoing implementation of clinical interventions at the organization, provider, and consumer levels.[22] Multiple implementation strategies have been identified that focus on increasing consumer engagement with EBPs, including: 1) increasing demand for EBPs, 2) intervening with consumers to enhance uptake and adherence, and 3) preparing consumers to be active participants in treatment.[23] These implementation strategies are consistent with evidence-based approaches to improve engagement in children's mental health care, which include assessment of barriers, accessibility promotion, psychoeducation about services, and appointment reminders.[24,25]

**Addressing Mental Health Service Disparities**

Lay Health Workers (LHWs) may be especially well positioned to deliver consumer-facing implementation strategies focused on addressing service disparities for underserved, low-resource communities.[26] LHWs, which include a range of terms, including *promotores*, family peer advocates, and wellness navigators, are individuals without formal mental health training, who have roles intended to increase their community's access to and benefit from services.[27,28] LHWs have the potential to address both demand and supply drivers of disparities in EBP delivery.[26] Demand for EBPs is impacted by an individual's mental health literacy, stigma towards mental illness and help seeking, perceptions of treatment providers, and culturally based beliefs and preferences.[29,30] Systemic barriers to care may exacerbate disparities in accessing care. For example, undocumented immigrants are especially unlikely to seek mental health services due to fear of being reported to authorities.[31] Since LHWs come from similar cultural

For peer review only

and personal backgrounds as the individuals they serve, they may be especially adept at helping patients overcome distrust of health systems.[32]

Regarding supply, the number of professional mental health providers who can deliver linguistically and culturally competent EBPs is inadequate.[33] The majority of mental health research with LHWs has been conducted in low- and middle-income countries, with emerging evidence that LHWs can improve mental health outcomes when they are tasked with delivering EBPs.[28,34,35] Though LHWs have successfully delivered BPTs as prevention interventions in high-income countries, using a task-shifting model,[36,37,38] licensure and certification requirements frequently restrict EBP delivery to professionals in mental health settings.[26] Therefore, LHWs in the United States may need to have complementary and distinct roles within the provision of EBPs.[11,26] For example, if LHWs delivered auxiliary engagement services (e.g., outreach and case management), it could reduce the burden on bilingual and bicultural mental health professionals and allow them to focus on activities that require advanced training and licensure, such as providing EBPs for more clients. [19,39]

One example of a LHW-delivered engagement program is the Parent Empowerment Program (PEP), which trains family peer advocates to work with parents to address their children's mental health needs and overcome barriers to care.[40,41] The majority of research on PEP has focused on evaluating the training of family peer advocates, as opposed to investigating the impact of the model on clinical outcomes for families, service utilization, or engagement in EBPs.[40–43] One randomized control trial evaluated the impact of PEP for Black and Latinx parents of children with autism. Parents who received PEP had significantly lower stress than parents who received treatment as usual. However, there were no group differences for service utilization. The researchers advocated that future research on programs like PEP should include

For peer review only

non-English-speaking families, who may have higher levels of need, and use qualitative research to better understand the strengths and areas of improvement for the model.[44] The proposed study follows these recommendations through the development and evaluation of LHWs Enhancing Engagement for Parents (LEEP), an implementation intervention to improve engagement for low-income, Latinx parents into one BPT, Parent-Child Interaction Therapy (PCIT).[45] LEEP seeks to follow recommendations by Chacko and colleagues (2016) based on their systematic review of parental engagement in BPTs by, "preparing parents for BPT, addressing practical barriers to engagement, assisting in aligning parent's involvement with their own goals for treatment" (p. 211) in order to impact initial and ongoing engagement in PCIT.

**Parent-Child Interaction Therapy**

PCIT has unique benefits and challenges related to engaging parents in treatment. The treatment model uses in vivo feedback to overcome challenges that are inherent to teaching methods used in other BPTs (e.g., didactics, discussion) as it necessitates active participation and assesses learning in real time. PCIT requires that parents demonstrate a high-level of proficiency with the targeted parenting skills before they advance from the first phase of treatment, which focuses on enhancing the parent-child relationship, to the second phase of treatment, which teaches effective and developmentally appropriate limit setting and discipline approaches, and then until they graduate from treatment.[45] Mastery-based criteria guarantees that all parents can successfully use the skills; however, parents often drop out before they learn the full range of parenting skills needed to decrease disruptive behaviors.[13,16] Furthermore, some research suggests that low-income, ethnic minority parents require more practice and time in treatment to reach this level of skill proficiency.[46–48] Extended treatment length can lead to long waitlists and fewer families seen in PCIT.[49]Further, problems with attendance, retention, and prolonged skill

acquisition have downstream effects on PCIT provider implementation outcomes. Clinicians can take up to three years to meet PCIT certification requirements (i.e., achieving fidelity and graduating two cases).[50] Thus, low parental engagement results in provider attrition from training, which in turn compromises the sustainability of the intervention, and limits the return on costly investments made to implement PCIT in public service systems.[8,9]

LEEP seeks to improve the supply of and demand for PCIT in agencies that predominately serve low-income, Latinx immigrant families, and address engagement challenges that impact clinical and implementation outcomes (Figure 1). PCIT is widely implemented in community settings, including in the county where the current study is being conducted. LEEP includes LHW-delivered implementation strategies to increase caregiver engagement as an extension of PCIT services, which will be provided by the licensed mental health professionals. LEEP will be compared to PCIT implementation-as-usual to see if parental engagement and implementation challenges are ameliorated. A community-partnered approach will focus on making LEEP a feasible and acceptable implementation intervention, with the following aims:

**Aim 1:** Assess the current context of LHW mobilization in children's mental health services, to inform the development of LEEP.

**Aim 2:** Through community-partnership, develop a structure for the implementation of LEEP in publicly-funded, children's mental health settings.

**Aim 3:** Evaluate the feasibility of implementing LEEP in community mental health agencies through a pilot effectiveness-implementation trial.

## Methods and Analysis

### Conceptual Framework and Approach

For peer review only - http://bmjopen.bmj.com/site/about/guidelines.xhtml

The Quality Implementation Framework (QIF), which includes four phases to support high quality implementation, informs the study aims and plans for scaling-up LEEP (Figure 2).[51] The first phase of the QIF focuses on assessing organizational needs, readiness, and innovation-organizational fit, which will be conducted in Aim 1 through survey data and stakeholder interviews. Phase 2 in the QIF focuses on the development of implementation structures, which will occur during the second aim with the input of a community-advisory group. The community-advisory group will collaboratively help to develop an implementation plan delineating tasks and timelines to establish infrastructure for LHW capacity building, including job descriptions and training plans. Phase 3 of the QIF includes three main activities that will take place during a hybrid type 2 effectiveness-implementation pilot stepped-wedge trial of LEEP. These activities include (1) providing implementation support strategies (e.g., supervision and consultation) to LHWs, (2) conducting a process evaluation to identify successes and barriers to implementation, and (3) providing ongoing feedback to organizations about the impact LEEP is having on service outcomes. Finally Aim 3 activities will inform Phase 4 of the QIF, which focuses on learning from initial implementation experiences to inform future efforts to scale-up and sustain LEEP.

**Patient and Public Involvement**

The research questions, study design, and outcomes measures were informed by public stakeholders, including agency leaders, PCIT therapists, and LHWs. No patients were involved in this process, though LHWs often have shared characteristics and life experiences given their social proximity to the individuals in the communities they serve.[52] The burden of the randomized control trial will be evaluated through the collection of feasibility and acceptability

For peer review only

data in qualitative interviews with participants. Results will be shared with participants in community-based events.

**Aim 1:** Assess the current context of LHW mobilization in children's mental health services, to inform the development of LEEP.

**Participants**. Surveys will be administered to LHWs employed or contracted by children's mental health agencies in two counties in California. Approximately 70 LHWs will be recruited to complete the quantitative survey. Based on a national survey of LHW,[53] LHW are expected to be Latinx, female, and have below a college level of education. Ten agency community mental health agency leaders and 25-30 LHW will be invited to participate in interviews to expand on the findings from the survey. Survey and interviews will be offered in Spanish or English.

**Procedure.** A mixed-method needs assessment will be conducted to understand how LHWs are currently mobilized in children's community mental health settings, with the purpose of adapting LEEP to fit within the local context. Surveys will provide a breadth of information and qualitative interviews will provide depth of information, to understand perceived barriers to parental engagement in children's mental health services, LHW roles and integration into services, and LHW knowledge about and attitudes towards BPTs and evidence-based engagement strategies. Data collection started in January 2017 and was completed in December 2018.

**Survey measures**. Surveys will be collected via electronic or paper-and-pencil survey based on LHW preferences.

***LHW characteristics***.[39] A demographic questionnaire will provide information about the LHWs' characteristics, including gender, race/ethnicity, country of origin, educational level, and

For peer review only

years of experience.

***Cultural background questionnaire.***[54] The Cultural Background Questionnaire is a 19-item self-report measure used to assess therapist generational status and acculturation, including cultural identity (i.e., U.S. identity and Heritage Cultural Identity) and language use.

***Parental engagement.***[55,56] A questionnaire that was developed to measure provider's perceptions of and strategies for engaging fathers has been adapted to measure LHW's perceptions of barriers to engagement, strategies for engagement, and confidence in engagement for parents. The adapted questionnaire includes perceived barriers in engagement for parents in general, and the LHW's use of and confidence with engagement strategies for both mothers and fathers.

***Attitudes towards BPT strategies.*** A four-item questionnaire was developed for this study to measure LHWs attitudes towards teaching parents common strategies targeted in BPTs including play to improve the parent-child relationship, praise of positive behaviors, ignoring minor misbehaviors, and time-out as a form of discipline.

***EBP questionnaire***.[57] A questionnaire developed to measure service broker's knowledge of and referrals to EBPs has been adapted to identify if LHWs are aware of, making referrals to, and supporting families involved specifically in PCIT(e.g., *"Have you referred parents to Parent-Child Interaction Therapy (PCIT)")*.

**Semi-Structured Interviews.** Interview guides will include topics, questions, and probes related to LHW roles, training needs, and experiences and attitudes related to BPTs. Questions will investigate how LHWs view their roles in agencies and their communities, their perceptions of BPTs, their preparation for their position, and their training needs. Interviews with agency leaders will focus on how LHW are integrated into services, LHW training, and outreach and

For peer review only

engagement strategies with Latinx families. A "funnel-approach" will be used with broad-open ended questions related to roles, trainings, and attitudes asked first, followed with specific probes to elicit details.[58]

**Analysis.** A QUAN + QUAL mixed methods design will be used, with quantitative and qualitative data collected simultaneously and given equal weight in analyses, for the purposes of gaining breadth and depth of understanding (i.e., complimentarity), identifying if the qualitative and quantitative data provide the same answer to the same question (i.e., convergence), and using qualitative data expand on unexpected quantitative findings.[59] Interviews will be transcribed and entered into *NVivo*, software that aids the coding, organization, and retrieval of codes. An iterative process will be used where the coding team first develops a preliminary coding scheme and applies it to a sample text to ensure all relevant themes are captured. Once a final coding scheme is decided upon, coders will apply the final code list to all transcripts. Regular meetings with the coding team will be conducted to examine coding across analysts, resolve differences in coding, conduct iterative refinement of code definitions and the logic of the coding tree, and collaborate on the development of themes. Qualitative themes will be identified through analysis of co-occurring codes and text analysis.[60,61]

**Aim 2:** Through community-partnership, develop a structure for the implementation of LEEP in children's mental health settings.

**Participants.** A community-advisory group with 6-9 stakeholders will be formed to make sure that implementation supports match the local context. Agency leaders, PCIT therapists, and LHWs will be represented in the advisory group. Given the wide diversity of viewpoints, education levels, and ethnicities, efforts will be made to provide each participant with equal representation, opportunities for contribution, and honorariums.

For peer review only

**Procedure.** In line with the Model of Research-Community Partnership, which was specifically developed for research in children's mental health services, the formation of the partnership will focus on building relationships, trust, establishing a joint mission, and identifying roles and responsibilities of different partner members. This will provide the foundation to build a synergistic, collaborative relationship focused on developing and delivering LEEP, which in turn could improve the successful and sustained implementation of PCIT.[62] Using data from Aim 1 and in collaboration with the community-advisory group, the LEEP implementation intervention will be adapted from an existing protocol focused on LHW-delivered parent outreach and engagement strategies. This protocol was developed to increase access to PCIT in a low-income, Latinx community in the Southeastern United States, but has not been disseminated to other communities.[39] The implementation supports needed for LHWs to deliver LEEP also will be identified and put into place. Steps from the QIF will be used to guide the activities of the community-advisory group in adapting these materials and developing LEEP's implementation structure.[51] Advisory group meetings will include: 1) activities to build trust and develop a shared mission statement, 2) feedback on adapting LEEP materials, 3) advisory group input on survey and interview results from Aim 1, and 4) development of a plan with specific tasks, roles, tracking for LEEP implementation. Phase 2 activities involving the community-advisory group began in December 2018 and will continue through March 2021.

**Aim 3:** Evaluate the feasibility of implementing LEEP through a pilot effectiveness-implementation trial.

In Aim 3, an effectiveness/ implementation hybrid design (Type 2) pilot study will integrate qualitative and quantitative data to examine the feasibility of delivering and scaling-up LEEP. Type 2 hybrid trials simultaneously measure the clinical effectiveness of an intervention,

in this case PCIT, and the feasibility and utility of an implementation intervention (i.e., LEEP).[63] Pilot studies are limited in their ability to test effectiveness given small sample sizes, but they provide a critical phase of research design that can examine the feasibility of the approach to be used in a large-scale study.[64] A focus will be placed on measuring engagement outcomes at a client- and agency-levels to evaluate if LEEP is increasing the reach of PCIT services, service entry, and treatment engagement.

**Procedure.** Three agency sites that provide PCIT will be involved in this pilot study, which will use a stepped wedge design. In a stepped wedge design, a period of baseline measurement will occur for all sites, in which PCIT will be implemented-as-usual. Then at subsequent time points each site will be randomized to LEEP and response to the intervention will be measured for client and implementation outcomes (Figure 3). At each agency, one to two LHWs (4-6 total) will be trained to deliver LEEP. Client and implementation outcomes will be collected during PCIT implementation-as-usual and LEEP implementation. The baseline measurement period began in January 2019. LHWs will be trained to deliver LEEP at the first site starting in July 2019 and will continue through March 2021.

**Participants.** Four to six LHWs will be trained to provide LEEP. These LHWs will provide LEEP care extension services to approximately PCIT clients each (16-24 families). LHWs will be trained to provide informed consent for parents. Families that participate in LEEP or PCIT implementation-as-usual will meet criteria for receiving this BPT. This includes having a child between the ages of 2 to 7 and presenting problems consistent with disruptive behaviors, or risk for child maltreatment.

**LEEP Intervention**. Based on community-advisory group feedback on the needs of the Latinx immigrant community and the agencies implementing PCIT, along with research on

For peer review only

parental engagement,[11] LEEP includes components for LHWs to 1) increase awareness of PCIT for Latinx immigrant families, 2) promote engagement once parents seek PCIT services, and 3) support parents' use of skills taught in PCIT throughout treatment. To increase knowledge of PCIT, LHWs will conduct community presentations in locations with parents of young children (e.g., Head Start Centers, churches). Parents will be referred to LEEP when they first seek services to promote engagement. LHWs will meet with parents in their home to discuss identify how PCIT aligns with their goals for treatment, address practical barriers to engagement (e.g., transportation), and introduce the relationship-enhancing parenting skills taught in PCIT. Once parents start PCIT, LHWs will provide home visits to promote skill practice and treatment adherence at the beginning of each treatment phase. Additional booster sessions will be provided based on the parent's progress in treatment. If parents have not reached mastery criteria after seven sessions, LHWs will conduct weekly home visits to reinforce skill use and address barriers to engagement. LHWs will be provided with electronic tablets with e-books that included scripts and videos to use in one-on-one meetings with parents before they enter into care and while they are receiving PCIT services. The e-books will have materials to help LHWs promote motivation (e.g., parent testimonials), homework adherence, and skill practice (e.g., video demonstrations of the targeted parenting skills).

**Effectiveness outcome measures.** Given that PCIT is an assessment-driven BPT, clinical outcomes will be assessed using standard measures that are collected as part of the routine PCIT protocol. Parents will not complete any additional measures for this study.

*Engagement.* To assess if LEEP impacts engagement at the family-level, session attendance, graduation from PCIT, and the number of sessions needed to graduate will be

For peer review only

assessed. Further, daily skill practice will be measured using the record sheets that parents complete over the week, which has been used in past studies on homework adherence.[65]

*The Dyadic Parent-Child Interaction Coding System (DPICS).*[66] The DPICS is a behavioral observation coding system that was designed to measure the quality of interaction in parent-child dyads, which has good interrater reliability. This study will use the DPICS categories, *Behavior Description, Labeled Praise, Unlabeled Praise, Reflection, Question, Negative Talk, and Indirect and Direct Commands,* to measure the parent's skill acquisition.

*Eyberg Child Behavior Inventory (ECBI).*[67] The ECBI is a 36-item parent-rating scale of disruptive behavior problems for children between the ages of 2 to 16. Parents rate the frequency of each disruptive behavior on a 7-point Likert scale ranging from *never* (1) to *always* (7), which are summed to yield the Intensity Scale and whether this behavior is a problem for them, with the total number of Yes responses yielding the Problem Scale.

**Implementation outcomes.** Using the implementation outcomes outlined by Proctor and colleagues,[68] this study uses mixed methods to understand the acceptability, appropriateness, feasibility, reach, and costs of delivering LEEP.

***LHW-level outcomes.*** To measure changes in LHW knowledge, perceptions of acceptability and feasibility of PCIT, and competence, LHW will complete pre- and post- self-report and behavioral measures (Table 1). Ongoing fidelity monitoring will be conducted throughout LHW's delivery of LEEP. Fidelity monitoring will include reviewing data capture of the ebook created for LEEP, which will include videos about PCIT and scripts for the LHWs to use with the families they serve. LHWs will also audio record their sessions to monitor fidelity to the LEEP model.

For peer review only

***Implementation costs.*** Costs associated with delivering LEEP will be measured by calculating time estimates associated with all aspects of implementation.

***Agency efficiencies***. To identify if LEEP impacts agency-efficiencies, with therapists increasing their billable hours, administrative claims will be calculated for PCIT therapists to measure the time spent in direct services.

***Reach and penetration.*** At the agency level, reach of PCIT will be assessed by the number of clients that enroll in and graduate from PCIT. Using administrative claims data, penetration at the agency-level will be calculated as the percentage of children receiving PCIT in out of the number of children who are eligible for this EBP. Furthermore, the percentage of families that successfully complete PCIT out of the families enrolled will be calculated.

***Acceptability and feasibility.*** Qualitative interviews will be conducted with the LHWs, agency leaders, and ten parents to assess their perceptions of LEEP including perceived acceptability, appropriateness, and feasibility, which are important early implementation outcomes.[68]

**Analysis.** This pilot trial is designed to evaluate the feasibility of implementing LEEP and develop tools to measure its clinical and implementation targets and outcomes. The trial is not powered to assess intervention effects. Analyses will focus on establishing the reliability and validity of measures of clinical engagement and implementation outcomes. Qualitative data will be analyzed using the methodology described in Aim 1. Qualitative and quantitative data will be given equal weight in analyses with a focus on *convergence*, *expansion*, and *complimentarity,* with quantitative data used to measure outcomes and qualitative data to understand process.[59]

## Discussion

For peer review only

LEEP has the potential for a significant public health impact, by developing an implementation intervention to increase entry and engagement of Latinx parents into BPTs to improve clinical and implementation outcomes. Though LHWs have been identified as an important workforce to address mental health disparities, limited research has evaluated the best strategies to mobilize them to support EBP implementation in the United States.[28] As a pilot study, findings will be limited in power and generalizability. However, the exploratory and development work in this study will provide data on the feasibility and acceptability of LEEP and its preliminary impact on client recruitment, adherence, and retention in PCIT, which will inform future scaling-up of the model.

**Ethics and dissemination:** Study procedures have been approved by the University of California, Santa Barbara IRB. Results will be submitted for publication in peer-reviewed journals. Furthermore, results will be shared with the community-advisory board and other stakeholders involved in the pilot of LEEP.

For peer review only

Table 1. Measures of LHW Training and Outcomes

| Measure | Description | Administration |
|---|---|---|
| *Demographic and Background Form*[39,54] | Characterizes personal and professional backgrounds of LHW | Aim 1: Survey<br>Aim 3: Training |
| *EBP Questionnaire*[57] | Measures service brokers' awareness of and referrals to EBPs. | Aim 1: Survey |
| *Parent Engagement Strategy Use and Confidence*[55,56] | Assess use and confidence of engagement strategies with mothers and fathers. | Aim 1: Survey<br>Aim 3: Pre-, Post-Training |
| *Acceptability and Feasibility of PCIT*[69] | Assess LHW perceptions of the acceptability and feasibility of PCIT | Aim 1: Survey<br>Aim 3: Pre-, Post-Training |
| *PCIT Knowledge Quiz* [39] | A quiz that measures the knowledge of PCIT principles and practices. | Aim 3: Pre-, Post-Training |
| *Dyadic Parent-Child Interaction Coding System*[66] | A behavioral observation coding system that assesses parent-child interactions. It will be used to measure LHW's ability to model parenting behaviors. | Aim 3: Pre-, Post-Training |
| *LEEP Fidelity Monitoring* | Review of ebook data capture to see the LEEP resources being used and audio recordings of home sessions. | Aim 3: During consultation |

For peer review only

## References


1. Fergusson DM, John Horwood L, Ridder EM. Show me the child at seven: the consequences of conduct problems in childhood for psychosocial functioning in adulthood. J Child Psychol Psychiatry. 2005 Aug;46(8):837–49. http://www.ncbi.nlm.nih.gov/pubmed/16033632

2. Rivenbark JG, Odgers CL, Caspi A, Harrington H, Hogan S, Houts RM, et al. The high societal costs of childhood conduct problems: evidence from administrative records up to age 38 in a longitudinal birth cohort. J Child Psychol Psychiatry. 2018 Jun 1;59(6):703–10. http://doi.wiley.com/10.1111/jcpp.12850

3. Prinz RJ, Sanders MR, Shapiro CJ, Whitaker DJ, Lutzker JR. Population-based prevention of child maltreatment: The U.S. Triple P system population trial. Prev Sci. 2016;17(3):410–6.

4. Chen M, Chan KL. Effects of parenting programs on child maltreatment prevention. Trauma, Violence, Abus. 2016 Jan 8;17(1):88–104. http://journals.sagepub.com/doi/10.1177/1524838014566718

5. Kaminski JW, Claussen AH. Evidence base update for psychosocial treatments for disruptive behaviors in children. J Clin Child Adolesc Psychol. 2017 Jul 4;46(4):477–99. https://www.tandfonline.com/doi/full/10.1080/15374416.2017.1310044

6. Miranda J, Bernal G, Lau A, Kohn L. State of the science on psychosocial interventions for ethnic minorities. Annu Rev Clin. 2005; http://www.annualreviews.org/doi/full/10.1146/annurev.clinpsy.1.102803.143822

7. Ortiz C, Del Vecchio T. Cultural diversity: Do we need a new wake-up call for parent training? Behav Ther. 2013 Sep;44(3):443–58. http://www.ncbi.nlm.nih.gov/pubmed/23768671

8. Beveridge RM, Fowles TR, Masse JJ, McGoron L, Smith MA, Parrish BP, et al. State-wide dissemination and implementation of parent-child interaction therapy (PCIT): Application of theory. Child Youth Serv Rev. 2015;48:38–48. http://dx.doi.org/10.1016/j.childyouth.2014.11.013

9. Timmer SG, Urquiza AJ, Boys DK, Forte LA, Quick-Abdullah D, Chan S, et al. Filling potholes on the implementation highway: Evaluating the implementation of Parent-Child Interaction Therapy in Los Angeles County. Child Abus Negl. 2016;53:40–50. http://dx.doi.org/10.1016/j.chiabu.2015.11.011

10. Baker CN, Arnold DH, Meagher S. Enrollment and attendance in a parent training prevention program for conduct problems. Prev Sci. 2011;12(2):126–38. http://www.springerlink.com/index/J410K861466J4204.pdf

11. Sanders MR. Triple P-Positive Parenting Program as a public health approach to strengthening parenting. J Fam Psychol. 2008;22(4):506–17.

12. Chacko A, Jensen SA, Lowry LS, Cornwell M, Chimklis A, Chan E, et al. Engagement in Behavioral Parent Training: Review of the Literature and Implications for Practice. Clin Child Fam Psychol Rev. 2016 Sep 16;19(3):204–15.


For peer review only

http://link.springer.com/10.1007/s10567-016-0205-2

13. Lanier P, Kohl PL, Benz J, Swinger D, Moussette P, Drake B. Parent-child interaction therapy in a community setting: Examining outcomes, attrition, and treatment setting. Res Soc Work Pract. 2011 Nov 4;21(6):689–98. http://journals.sagepub.com/doi/10.1177/1049731511406551

14. Budd KS, Lyon AR. A community mental health implementation of Parent-Child Interaction Therapy (PCIT). J Child Fam Stud. 2010 Oct 3;19(5):654–68. http://link.springer.com/10.1007/s10826-010-9353-z

15. Pearl E, Thieken L, Olafson E, Boat B, Connelly L, Barnes J, et al. Effectiveness of community dissemination of parent-child interaction therapy. Psychol Trauma Theory, Res Pract Policy. 2012;4(2):204–13. https://bobcat.militaryfamilies.psu.edu/sites/default/files/placed-programs/Pearl et al 2012 effectiveness of community dissemination.pdf

16. Boggs SR, Eyberg SM, Edwards DL, Rayfield A, Jacobs J, Bagner D, et al. Outcomes of parent-child interaction therapy: A comparison of treatment completers and study dropouts one to three years later. Child Fam Behav Ther. 2005;26(4):1–22. http://www.tandfonline.com/action/journalInformation?journalCode=wcfb20

17. Hoagwood KE, Olin SS, Horwitz S, McKay M, Cleek A, Gleacher A, et al. Scaling up evidence-based practices for children and families in New York State: Toward evidence-based policies on implementation for state mental health systems. J Clin Child Adolesc Psychol. 2014 Mar 24;43(2):145–57. http://www.tandfonline.com/doi/abs/10.1080/15374416.2013.869749

18. Stewart RE, Adams DR, Mandell DS, Hadley TR, Evans AC, Rubin R, et al. The perfect storm: Collision of the business of mental health and the implementation of evidence-based practices. Psychiatr Serv. 2016;67(2):159–61. www.ctacny.com/beep-business-

19. Regan J, Lau AS, Barnett M, Stadnick N, Hamilton A, Pesanti K, et al. Agency responses to a system-driven implementation of multiple evidence-based practices in children's mental health services. BMC Health Serv Res. 2017;17(1).

20. Alegria M, Vallas M, Pumariega A. Racial and ethnic disparities in pediatric mental health. Child Adolesc Psychiatr. 2010; http://www.sciencedirect.com/science/article/pii/S1056499310000568

21. Coker TR, Elliott MN, Kataoka S, Schwebel DC, Mrug S, Grunbaum JA, et al. Racial/ethnic disparities in the mental health care utilization of fifth grade children. Acad Pediatr. 2009 Mar 1;9(2):89–96. https://www.sciencedirect.com/science/article/pii/S1876285908002672

22. Eldh AC, Almost J, Decorby-Watson K, Gifford W, Harvey G, Hasson H, et al. Clinical interventions, implementation interventions, and the potential greyness in between -a discussion paper [Internet]. Vol. 17, BMC Health Services Research. BioMed Central; 2017 [cited 2018 Oct 28]. p. 16. http://www.ncbi.nlm.nih.gov/pubmed/28061856

23. Powell BJ, Waltz TJ, Chinman MJ, Damschroder LJ, Smith JL, Matthieu MM, et al. A

For peer review only

refined compilation of implementation strategies: Results from the Expert Recommendations for Implementing Change (ERIC) project. Implement Sci. 2015 Dec 12;10(1):21. http://implementationscience.biomedcentral.com/articles/10.1186/s13012-015-0209-1

24. Becker KD, Lee BR, Daleiden EL, Lindsey M, Brandt NE, Chorpita BF. The common elements of engagement in children's mental health services: Which elements for which outcomes? J Clin Child Adolesc Psychol. 2015;44(1):30–43.

25. Becker KD, Boustani M, Gellatly R, Chorpita BF. Forty years of engagement research in children's mental health services: Multidimensional measurement and practice elements. J Clin Child Adolesc Psychol. 2018 Jan 2;47(1):1–23. https://www.tandfonline.com/doi/full/10.1080/15374416.2017.1326121

26. Barnett ML, Lau AS, Miranda J. Lay health worker involvement in evidence-based treatment delivery: A conceptual model to address disparities in care. Annu Rev Clin Psychol. 2018 May 7;14(1):185–208. http://www.annualreviews.org/doi/10.1146/annurev-clinpsy-050817-084825

27. Ayala G, Vaz L, Earp J, Elder J. Outcome effectiveness of the lay health advisor model among Latinos in the United States: an examination by role. Health Educ. 2010; https://academic.oup.com/her/article-abstract/25/5/815/567914

28. Barnett ML, Gonzalez A, Miranda J, Chavira DA, Lau AS. Mobilizing community health workers to address mental health disparities for underserved populations: A systematic review. Adm Policy Ment Heal Ment Heal Serv Res. 2018;45(2):195–211.

29. Alegria M, Vallas M, Pumariega A, Becker KD, Lee BR, Daleiden EL, et al. NIH Public Access. Child Youth Serv Rev. 2015;44(2):181–217. http://www.sciencedirect.com/science/article/pii/S0190740915001085

30. Kilbourne AM, Switzer G, Hyman K, Crowley-Matoka M, Fine MJ. Advancing health disparities research within the health care system: A conceptual framework. Am J Public Health. 2006 Dec;96(12):2113–21. http://www.ncbi.nlm.nih.gov/pubmed/17077411

31. Philbin MM, Flake M, Hatzenbuehler ML, Hirsch JS. State-level immigration and immigrant-focused policies as drivers of Latino health disparities in the United States. Soc Sci Med. 2018 Feb 1;199:29–38. https://www.sciencedirect.com/science/article/pii/S0277953617302307

32. Katigbak C, Van Devanter N, Islam N T-S. Partners in health: A conceptual framework for the role of community health workers in facilitating patients' adoption of healthy behaviors. J Public …. 2015; https://ajph.aphapublications.org/doi/full/10.2105/AJPH.2014.302411

33. Huang L, MacBeth G, Dodge J, Jacobstein D. Transforming the workforce in children's mental health [Internet]. Vol. 32, Administration and Policy in Mental Health. 2004 [cited 2018 Nov 5]. p. 167–87. https://link.springer.com/content/pdf/10.1023/B:APIH.0000042745.64582.72.pdf

34. Singla DR, Kohrt BA, Murray LK, Anand A, Chorpita BF, Patel V. Psychological

For peer review only

Treatments for the World: Lessons from Low- and Middle-Income Countries. Annu Rev Clin Psychol. 2017;13(1):149–81. http://www.annualreviews.org/doi/10.1146/annurev-clinpsy-032816-045217

35. Nadja : van Ginneken, Prathap : Tharyan, Simon : Lewin, N : Rao Girish, SM : Meera, Jessica : Pian, et al. Non-specialist health worker interventions for the care of mental, neurological and substance-abuse disorders in low- and middle-income countries SO-: Cochrane Database of Systematic Reviews YR-: 2013 NO-: 11. 2013;(11).

36. Chacko A, Scavenius C. Bending the Curve: A Community-Based Behavioral Parent Training Model to Address ADHD-Related Concerns in the Voluntary Sector in Denmark. J Abnorm Child Psychol. 2018 Apr 23;46(3):505–17. http://link.springer.com/10.1007/s10802-017-0310-9

37. Chacko A, Fabiano GA, Doctoroff GL, Fortson B. Engaging Fathers in Effective Parenting for Preschool Children Using Shared Book Reading: A Randomized Controlled Trial. J Clin Child Adolesc Psychol. 2018 Jan 2;47(1):79–93. http://www.ncbi.nlm.nih.gov/pubmed/28103110

38. Williamson AA, Knox L, Guerra NG, Williams KR. A Pilot Randomized Trial of Community-Based Parent Training for Immigrant Latina Mothers. Am J Community Psychol. 2014 Mar;53(1–2):47–59. http://doi.wiley.com/10.1007/s10464-013-9612-4

39. Barnett ML, Davis EM, Callejas LM, White JV, Acevedo-Polakovich ID, Niec LN, et al. The development and evaluation of a natural helpers' training program to increase the engagement of urban, Latina/o families in parent-child interaction therapy. Child Youth Serv Rev. 2016;65.

40. Rodriguez J, Olin SS, Hoagwood KE, Shen S, Burton G, Radigan M, et al. The Development and Evaluation of a Parent Empowerment Program for Family Peer Advocates. J Child Fam Stud. 2011 Aug;20(4):397–405. http://www.ncbi.nlm.nih.gov/pubmed/25382959

41. Olin SS, Hoagwood KE, Rodriguez J, Radigan M, Burton G, Cavaleri M, et al. Impact of Empowerment Training on the Professional Work of Family Peer Advocates. Child Youth Serv Rev. 2010 Oct 1;32(10):1426–9. http://www.ncbi.nlm.nih.gov/pubmed/21076659

42. Olin SS, Williams N, Pollock M, Armusewicz K, Kutash K, Glisson C, et al. Quality Indicators for Family Support Services and Their Relationship to Organizational Social Context. Adm Policy Ment Heal Ment Heal Serv Res. 2014 Jan 25;41(1):43–54. http://link.springer.com/10.1007/s10488-013-0499-z

43. Hoagwood KE, Olin SS, Storfer-Isser A, Kuppinger A, Shorter P, Wang NM, et al. Evaluation of a Train-The-Trainers Model for Family Peer Advocates in Children's Mental Health. J Child Fam Stud. 2018 Apr 1;27(4):1130–6. http://link.springer.com/10.1007/s10826-017-0961-8

44. Jamison JM, Fourie E, Siper PM, Trelles MP, George-Jones J, Buxbaum Grice A, et al. Examining the efficacy of a family peer advocate model for Black and Hispanic caregivers of children with Autism Spectrum Disorder. J Autism Dev Disord. 2017 May 6;47(5):1314–22. http://link.springer.com/10.1007/s10803-017-3045-0

For peer review only

45. Funderburk BW, Eyberg S. Parent–child interaction therapy. In: History of psychotherapy: Continuity and change (2nd ed). Washington: American Psychological Association; p. 415–20. http://content.apa.org/books/12353-021

46. McCabe K, Yeh M. Parent-child interaction therapy for Mexican Americans: A randomized clinical trial. J Clin Child Adolesc Psychol. 2009 Aug 31;38(5):753–9. http://www.ncbi.nlm.nih.gov/pubmed/20183659

47. Bernal G. Parent-Child Interaction Therapy for Puerto Rican preschool children with ADHD and behavior Problems : A pilot efficacy study. Fam Process. 2009;48(2):232–52. www.FamilyProcess.org

48. Ramos G, Blizzard AM, Barroso NE, Bagner DM. Parent training and skill acquisition and utilization among Spanish- and English-speaking Latino families. J Child Fam Stud. 2018 Jan 17;27(1):268–79. http://link.springer.com/10.1007/s10826-017-0881-7

49. Gross D, Belcher HME, Budhathoki C, Ofonedu ME, Uveges MK. Does Parent Training Format Affect Treatment Engagement? A Randomized Study of Families at Social Risk. J Child Fam Stud. 2018 May 29;27(5):1579–93. http://link.springer.com/10.1007/s10826-017-0984-1

50. Scudder AT, Herschell AD. Building an evidence-base for the training of evidence-based treatments in community settings: Use of an expert-informed approach. Child Youth Serv Rev. 2015;55:84–92. http://dx.doi.org/10.1016/j.childyouth.2015.05.003

51. Meyers DC, Durlak JA, Wandersman A. The Quality Implementation Framework: A synthesis of critical steps in the implementation process. Am J Community Psychol. 2012;50(3–4):462–80.

52. Gustafson EL, Atkins M, Rusch D. Community health workers and social proximity: Implementation of a parenting program in urban poverty. Am J Community Psychol. 2018 Dec 1;62(3–4):449–63. http://doi.wiley.com/10.1002/ajcp.12274

53. Ingram M, Reinschmidt KM, Schachter KA, Davidson CL, Sabo SJ, De Zapien JG, et al. Establishing a professional profile of Community Health Workers: Results from a national study of roles, activities and training. J Community Health. 2012;37(2):529–37.

54. Saifan D, Brookman-Frazee L, Barnett M, Gonzalez JC, Lau AS. Ethnic minority community therapists' acculturation and reported adaptations to children's evidence-based practices. Cult Divers Ethn Minor Psychol. 2018;24(4):530–40.

55. Tully LA, Collins DAJ, Piotrowska PJ, Mairet KS, Hawes DJ, Moul C, et al. Examining practitioner competencies, organizational support and barriers to engaging fathers in parenting interventions. Child Psychiatry Hum Dev. 2018;49(1):109–22. http://www.ncbi.nlm.nih.gov/pubmed/28523378

56. Jiang Y, Tully LA, Burn MT, Piotrowska P, Collins DAJ, Moul C, et al. Development and psychometric evaluation of the father engagement questionnaire. J Child Fam Stud. 2018 Jul 20;27(11):3457–67. http://link.springer.com/10.1007/s10826-018-1195-0

57. Dorsey S, Kerns SEU, Trupin EW, Conover KL, Berliner L. Child welfare caseworkers as service brokers for youth in foster care: Findings from project focus. Child Maltreat.

For peer review only

2012;17(1):22–31.

58. Spradley J. The Ethnographic Interview. The Ethnographic Interview. Long Grove, IL: Waveland Press, Inc.; 1979.

59. Palinkas LA, Aarons GA, Horwitz S, Chamberlain P, Hurlburt M, Landsverk J. Mixed method designs in implementation research. Adm Policy Ment Heal Ment Heal Serv Res. 2011;38(1):44–53.

60. Bradley EH, Curry LA, Devers KJ. Qualitative data analysis for health services research: Developing taxonomy, themes, and theory. Health Serv Res. 2007 Aug;42(4):1758–72. http://www.ncbi.nlm.nih.gov/pubmed/17286625

61. Palinkas LA. Qualitative and mixed methods in mental health services and implementation research. J Clin Child Adolesc Psychol. 2014 Nov 2;43(6):851–61. http://www.ncbi.nlm.nih.gov/pubmed/25350675

62. Brookman-Frazee L, Stahmer A, Stadnick N, Chlebowski C, Herschell A, Garland AF. Characterizing the use of research-community partnerships in studies of evidence-based Interventions in children's community services. Adm Policy Ment Heal Ment Heal Serv Res. 2016;43(1):93–104.

63. Curran GM, Bauer M, Mittman B, Pyne JM, Stetler C. Effectiveness-implementation hybrid designs. Med Care. 2012 Mar;50(3):217–26. http://www.ncbi.nlm.nih.gov/pubmed/22310560

64. Leon AC, Davis LL, Kraemer HC. Role and interpretation of pilot studies in clinical research. J Psychiatr Res. 2012;45(5):626–9.

65. Stokes JO, Jent JF, Weinstein A, Davis EM, Brown TM, Cruz L, et al. Does Practice Make Perfect? The Relationship Between Self-Reported Treatment Homework Completion and Parental Skill Acquisition and Child Behaviors. Behav Ther. 2016 Jul 1;47(4):538–49. https://www.sciencedirect.com/science/article/abs/pii/S0005789416300120

66. Robinson EA, Eyberg SM. The dyadic parent–child interaction coding system: Standardization and validation. J Consult Clin Psychol. 1981;49(2):245–50. http://doi.apa.org/getdoi.cfm?doi=10.1037/0022-006X.49.2.245

67. Boggs SR, Eyberg S, Ann Reynolds L. Concurrent Validity of the Eyberg Child Behavior Inventory. J Clin Child Psychol. 1990;19(1):75–8. https://www.tandfonline.com/action/journalInformation?journalCode=hcap20

68. Proctor E, Silmere H, Raghavan R, Hovmand P, Aarons G, Bunger A, et al. Outcomes for implementation research: Conceptual distinctions, measurement challenges, and research agenda. Adm Policy Ment Heal Ment Heal Serv Res. 2011 Mar;38(2):65–76. http://www.ncbi.nlm.nih.gov/pubmed/20957426

69. Weiner BJ, Lewis CC, Stanick C, Powell BJ, Dorsey CN, Clary AS, et al. Psychometric assessment of three newly developed implementation outcome measures. Implement Sci. 2017 Dec 29;12(1):108. http://implementationscience.biomedcentral.com/articles/10.1186/s13012-017-0635-3

For peer review only

**Author Statement**

MLB is the principal investigator for the study protocol. MLB generated the idea and design of the study, and was the primary writer of the manuscript. ASL, JM, JL, MKK, and LS also made substantial contributions to study conception and design as mentors to MLB for this K01 award. All authors reviewed and provided feedback for this manuscript. The final version of this manuscript was vetted and approved by all authors.

**Funding**

This study was funded by the National Institute of Mental Health (K01MH110608) awarded to MLB. Additionally, the preparation of this article was supported in part by the Implementation Research Institute (IRI) at the Brown School, Washington University in St. Louis, through an award from the National Institute of Mental Health (R25 MH080916). MLB is a fellow of IRI.

**Competing interests**

None declared

**Ethics Approval**

University of California, Santa Barbara IRB approved study procedures (Protocol number: 1-18-0919 and 4-19-0167).

**Acknowledgements**

The authors would like to thank Camille Nebeker and Emily Winslow, who serve as consultants on this project. We are grateful to the help provided by Juan Carlos Gonzalez, Corinna Klein, Erika Luis Sanchez, Iliana Flores, and Ana Romero, who assisted on this project. Finally, we would like to thank our community partners at the Santa Barbara Promotora Network and CALM (Child Abuse Mediation and Listening).

For peer review only

Figure 1. LEEP's Approach to Address Supply and Demand Determinants of Disparities

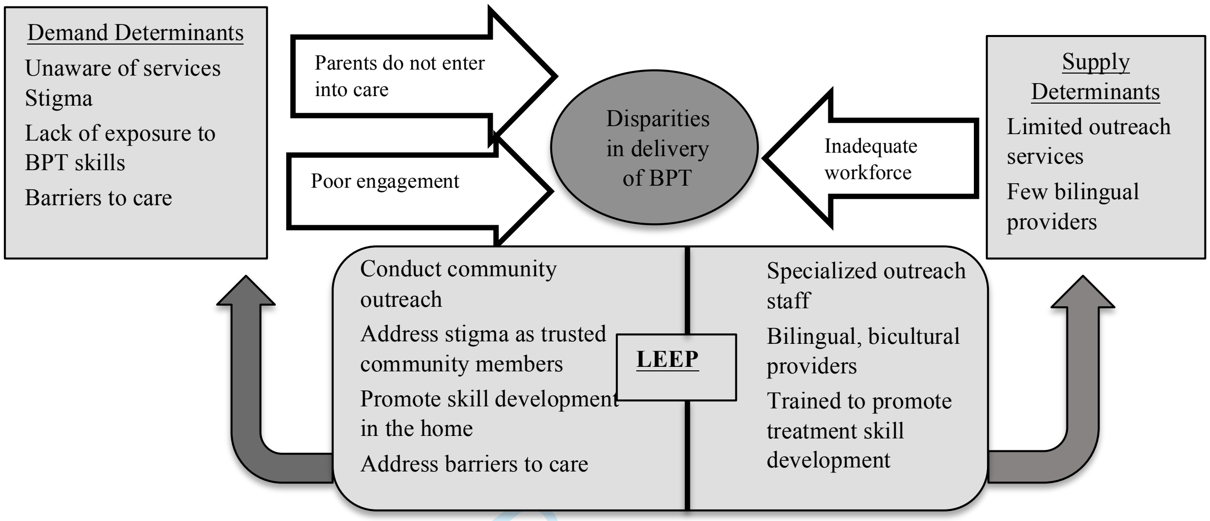


Adapted from Barnett, Lau, & Miranda, 2018. LEEP seeks to address supply and demand drivers of demand for BPTs.

For peer review only

Figure 2. Critical Steps of QIF in LEEP Development and Implementation

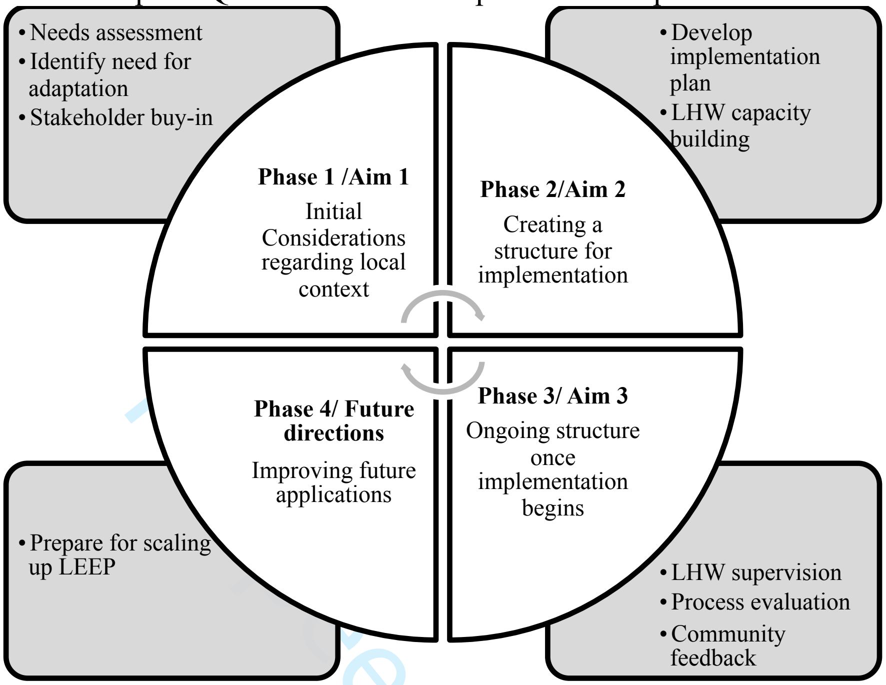


Adapted from Meyers et al. (2012). The steps of the QIF inform each aim of the pilot study and plans for scaling.

For peer review only

Figure 3. Stepped Wedge Trial of LEEP Implementation

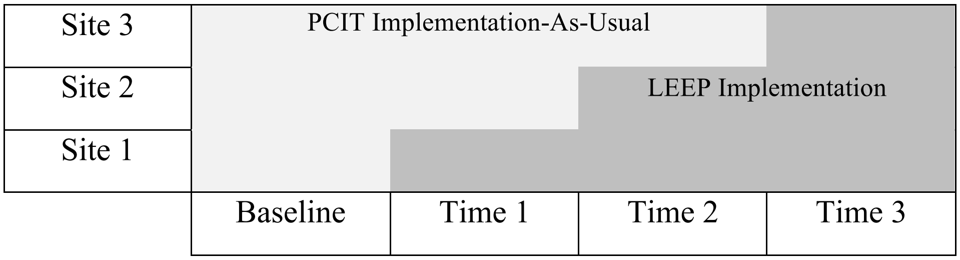


A stepped-wedge design will be used with the three sites implementing LEEP at separate time points.

For peer review only

# Reporting checklist for protocol of a clinical trial.

Based on the SPIRIT guidelines.

## Instructions to authors

Complete this checklist by entering the page numbers from your manuscript where readers will find each of the items listed below.

Your article may not currently address all the items on the checklist. Please modify your text to include the missing information. If you are certain that an item does not apply, please write "n/a" and provide a short explanation.

Upload your completed checklist as an extra file when you submit to a journal.

In your methods section, say that you used the SPIRIT reporting guidelines, and cite them as:

Chan A-W, Tetzlaff JM, Altman DG, Laupacis A, Gøtzsche PC, Krleža-Jerić K, Hróbjartsson A, Mann H, Dickersin K, Berlin J, Doré C, Parulekar W, Summerskill W, Groves T, Schulz K, Sox H, Rockhold FW, Rennie D, Moher D. SPIRIT 2013 Statement: Defining standard protocol items for clinical trials. Ann Intern Med. 2013;158(3):200-207

| | | Reporting Item | Page Number |
|---|---|---|---|
| Title | #1 | Descriptive title identifying the study design, population, interventions, and, if applicable, trial acronym | 1 |
| Trial registration | #2a | Trial identifier and registry name. If not yet registered, name of intended registry | N/A |
| Trial registration: data set | #2b | All items from the World Health Organization Trial Registration Data Set | N/A |
| Protocol version | #3 | Date and version identifier | N/A |
| Funding | #4 | Sources and types of financial, material, and other support | 27 |
| Roles and responsibilities: contributorship | #5a | Names, affiliations, and roles of protocol contributors | 27 |
| Roles and responsibilities: | #5b | Name and contact information for the trial sponsor | 1 |

For peer review only

| | | | |
|---|---|---|---|
| sponsor contact information | | | |
| Roles and responsibilities: sponsor and funder | #5c | Role of study sponsor and funders, if any, in study design; collection, management, analysis, and interpretation of data; writing of the report; and the decision to submit the report for publication, including whether they will have ultimate authority over any of these activities | 28 |
| Roles and responsibilities: committees | #5d | Composition, roles, and responsibilities of the coordinating centre, steering committee, endpoint adjudication committee, data management team, and other individuals or groups overseeing the trial, if applicable (see Item 21a for data monitoring committee) | 13 |
| Background and rationale | #6a | Description of research question and justification for undertaking the trial, including summary of relevant studies (published and unpublished) examining benefits and harms for each intervention | 4-9 |
| Background and rationale: choice of comparators | #6b | Explanation for choice of comparators | 9 |
| Objectives | #7 | Specific objectives or hypotheses | 9 |
| Trial design | #8 | Description of trial design including type of trial (eg, parallel group, crossover, factorial, single group), allocation ratio, and framework (eg, superiority, equivalence, non-inferiority, exploratory) | 10 |
| Study setting | #9 | Description of study settings (eg, community clinic, academic hospital) and list of countries where data will be collected. Reference to where list of study sites can be obtained | 10 |
| Eligibility criteria | #10 | Inclusion and exclusion criteria for participants. If applicable, eligibility criteria for study centres and individuals who will perform the interventions (eg, surgeons, psychotherapists) | 15 |
| Interventions: description | #11a | Interventions for each group with sufficient detail to allow replication, including how and when they will be administered | 15 |
| Interventions: modifications | #11b | Criteria for discontinuing or modifying allocated interventions for a given trial participant (eg, drug dose change in response to harms, participant request, or improving / worsening disease) | N/A |
| Interventions: adherance | #11c | Strategies to improve adherence to intervention protocols, and any procedures for monitoring adherence (eg, drug tablet return; | 16 |

For peer review only

| | | | |
|---|---|---|---|
| | | laboratory tests) | |
| Interventions: concomitant care | #11d | Relevant concomitant care and interventions that are permitted or prohibited during the trial | N/A |
| Outcomes | #12 | Primary, secondary, and other outcomes, including the specific measurement variable (eg, systolic blood pressure), analysis metric (eg, change from baseline, final value, time to event), method of aggregation (eg, median, proportion), and time point for each outcome. Explanation of the clinical relevance of chosen efficacy and harm outcomes is strongly recommended | 16 |
| Participant timeline | #13 | Time schedule of enrolment, interventions (including any run-ins and washouts), assessments, and visits for participants. A schematic diagram is highly recommended (see Figure) | 14 |
| Sample size | #14 | Estimated number of participants needed to achieve study objectives and how it was determined, including clinical and statistical assumptions supporting any sample size calculations | 14 |
| Recruitment | #15 | Strategies for achieving adequate participant enrolment to reach target sample size | 14 |
| Allocation: sequence generation | #16a | Method of generating the allocation sequence (eg, computer-generated random numbers), and list of any factors for stratification. To reduce predictability of a random sequence, details of any planned restriction (eg, blocking) should be provided in a separate document that is unavailable to those who enrol participants or assign interventions | N/A |
| Allocation concealment mechanism | #16b | Mechanism of implementing the allocation sequence (eg, central telephone; sequentially numbered, opaque, sealed envelopes), describing any steps to conceal the sequence until interventions are assigned | N/A |
| Allocation: implementation | #16c | Who will generate the allocation sequence, who will enrol participants, and who will assign participants to interventions | N/A |
| Blinding (masking) | #17a | Who will be blinded after assignment to interventions (eg, trial participants, care providers, outcome assessors, data analysts), and how | N/A |
| Blinding (masking): emergency | #17b | If blinded, circumstances under which unblinding is permissible, and procedure for revealing a participant's allocated intervention during | N/A |

For peer review only

| | | | |
|---|---|---|---|
| unblinding | | the trial | |
| Data collection plan | #18a | Plans for assessment and collection of outcome, baseline, and other trial data, including any related processes to promote data quality (eg, duplicate measurements, training of assessors) and a description of study instruments (eg, questionnaires, laboratory tests) along with their reliability and validity, if known. Reference to where data collection forms can be found, if not in the protocol | 15-16 |
| Data collection plan: retention | #18b | Plans to promote participant retention and complete follow-up, including list of any outcome data to be collected for participants who discontinue or deviate from intervention protocols | N/A |
| Data management | #19 | Plans for data entry, coding, security, and storage, including any related processes to promote data quality (eg, double data entry; range checks for data values). Reference to where details of data management procedures can be found, if not in the protocol | N/A |
| Statistics: outcomes | #20a | Statistical methods for analysing primary and secondary outcomes. Reference to where other details of the statistical analysis plan can be found, if not in the protocol | 16 |
| Statistics: additional analyses | #20b | Methods for any additional analyses (eg, subgroup and adjusted analyses) | N/A |
| Statistics: analysis population and missing data | #20c | Definition of analysis population relating to protocol non-adherence (eg, as randomised analysis), and any statistical methods to handle missing data (eg, multiple imputation) | N/A |
| Data monitoring: formal committee | #21a | Composition of data monitoring committee (DMC); summary of its role and reporting structure; statement of whether it is independent from the sponsor and competing interests; and reference to where further details about its charter can be found, if not in the protocol. Alternatively, an explanation of why a DMC is not needed | N/A |
| Data monitoring: interim analysis | #21b | Description of any interim analyses and stopping guidelines, including who will have access to these interim results and make the final decision to terminate the trial | N/A |
| Harms | #22 | Plans for collecting, assessing, reporting, and managing solicited and spontaneously reported adverse events and other unintended effects of trial interventions or trial conduct | N/A |
| Auditing | #23 | Frequency and procedures for auditing trial conduct, if any, and | N/A |

For peer review only

| | | | |
|---|---|---|---|
| | | whether the process will be independent from investigators and the sponsor | |
| Research ethics approval | #24 | Plans for seeking research ethics committee / institutional review board (REC / IRB) approval | 18 |
| Protocol amendments | #25 | Plans for communicating important protocol modifications (eg, changes to eligibility criteria, outcomes, analyses) to relevant parties (eg, investigators, REC / IRBs, trial participants, trial registries, journals, regulators) | N/A |
| Consent or assent | #26a | Who will obtain informed consent or assent from potential trial participants or authorised surrogates, and how (see Item 32) | 14 |
| Consent or assent: ancillary studies | #26b | Additional consent provisions for collection and use of participant data and biological specimens in ancillary studies, if applicable | N/A |
| Confidentiality | #27 | How personal information about potential and enrolled participants will be collected, shared, and maintained in order to protect confidentiality before, during, and after the trial | N/A |
| Declaration of interests | #28 | Financial and other competing interests for principal investigators for the overall trial and each study site | 27 |
| Data access | #29 | Statement of who will have access to the final trial dataset, and disclosure of contractual agreements that limit such access for investigators | N/A |
| Ancillary and post trial care | #30 | Provisions, if any, for ancillary and post-trial care, and for compensation to those who suffer harm from trial participation | N/A |
| Dissemination policy: trial results | #31a | Plans for investigators and sponsor to communicate trial results to participants, healthcare professionals, the public, and other relevant groups (eg, via publication, reporting in results databases, or other data sharing arrangements), including any publication restrictions | 18 |
| Dissemination policy: authorship | #31b | Authorship eligibility guidelines and any intended use of professional writers | 27 |
| Dissemination policy: reproducible research | #31c | Plans, if any, for granting public access to the full protocol, participant-level dataset, and statistical code | N/A |
| Informed consent materials | #32 | Model consent form and other related documentation given to participants and authorised surrogates | Suppl |

For peer review only

| | | | |
|---|---|---|---|
| Biological specimens | #33 | Plans for collection, laboratory evaluation, and storage of biological specimens for genetic or molecular analysis in the current trial and for future use in ancillary studies, if applicable | N/A |

The SPIRIT checklist is distributed under the terms of the Creative Commons Attribution License CC-BY-ND 3.0. This checklist was completed on 07. January 2019 using https://www.goodreports.org/, a tool made by the EQUATOR Network in collaboration with Penelope.ai

For peer review only
